# Supplementary material for: First Trimester Prediction of Preterm Delivery in the Absence of Other Pregnancy-Related Complications Using Cardiovascular-Disease Associated MicroRNA Biomarkers
Source: Int J Mol Sci. 2022 Apr 1;23(7):3951. doi: 10.3390/ijms23073951 (PMC8999783; doi:10.3390/ijms23073951)
Supplement: Supplementary file 1 [file ijms-23-03951-s001.zip › Supplementary Figure S1.pdf]

Supplementary Figure S1.

A

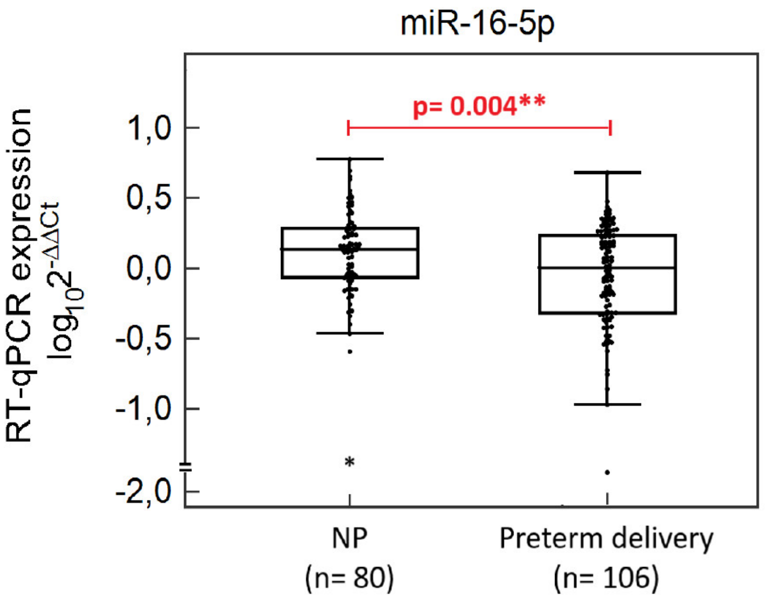

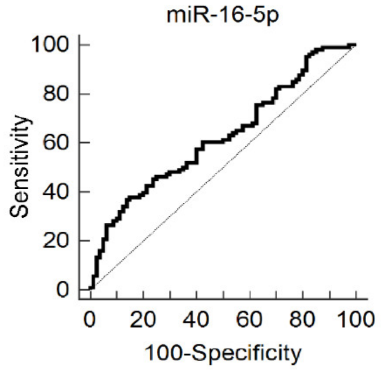

Area under the ROC curve (AUC)	0.625
Standard Error	0.0409
95% Confidence interval	0.551 to 0.695
Significance level P (Area=0.5)	0.0022

Estimated sensitivity at fixed specificity			
Specificity	Sensitivity	95% CI	Criterion
90.00	29.25	14.15 to 41.51	≤0.597445814

Criterion	Sensitivity	95% CI	Specificity	95% CI	+LR	95% CI	-LR	95% CI
≤0.70928317	36.79	27.6 - 46.7	86.25	76.7 - 92.9	2.68	1.5 - 4.9	0.73	0.6 - 0.9

B

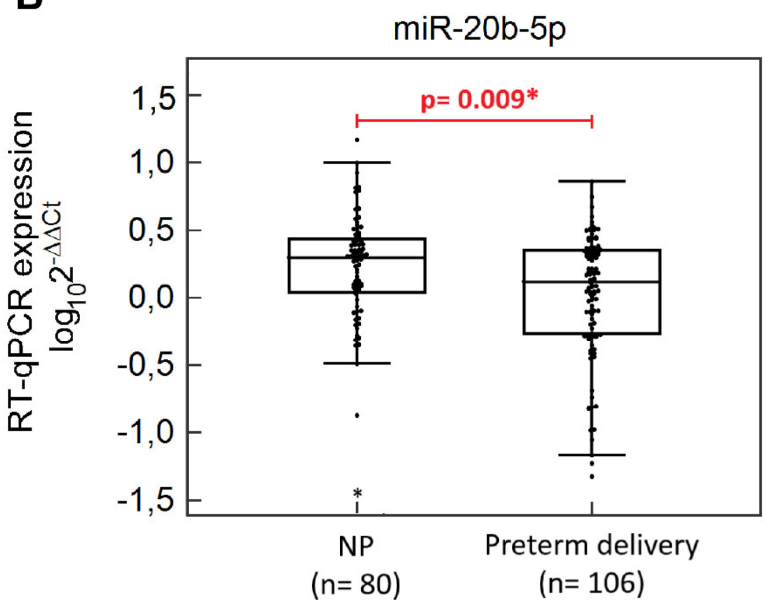

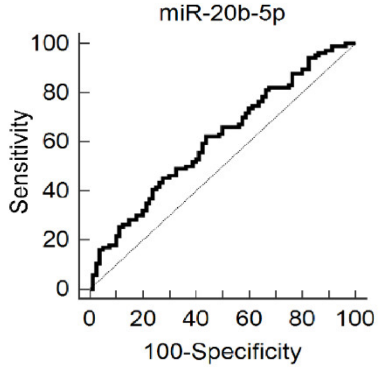

Area under the ROC curve (AUC)	0.612
Standard Error	0.0415
95% Confidence interval	0.537 to 0.682
Significance level P (Area=0.5)	0.0072

Estimated sensitivity at fixed specificity			
Specificity	Sensitivity	95% CI	Criterion
90.00	21.70	10.38 to 34.91	≤0.531505672

Criterion	Sensitivity	95% CI	Specificity	95% CI	+LR	95% CI	-LR	95% CI
≤1.66069881	62.26	52.3 - 71.5	56.25	44.7 - 67.3	1.42	1.1 - 1.9	0.67	0.5 - 0.9

C

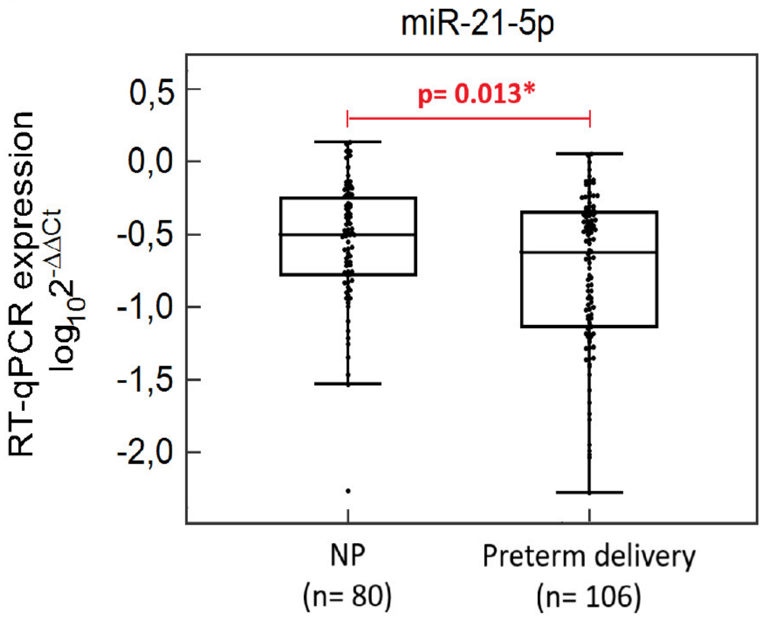

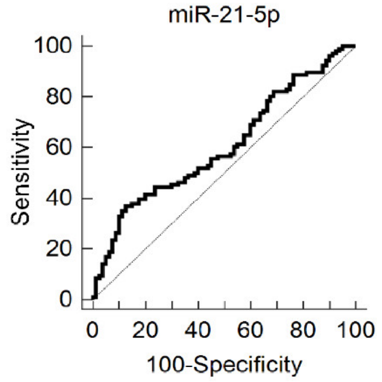

Area under the ROC curve (AUC)	0.606
Standard Error	0.0414
95% Confidence interval	0.532 to 0.677
Significance level P (Area=0.5)	0.0102

Estimated sensitivity at fixed specificity			
Specificity	Sensitivity	95% CI	Criterion
90.00	33.02	16.04 to 47.17	≤0.100086787

Criterion	Sensitivity	95% CI	Specificity	95% CI	+LR	95% CI	-LR	95% CI
≤0.115689703	36.79	27.6 - 46.7	87.50	78.2 - 93.8	2.94	1.6 - 5.5	0.72	0.6 - 0.9

D

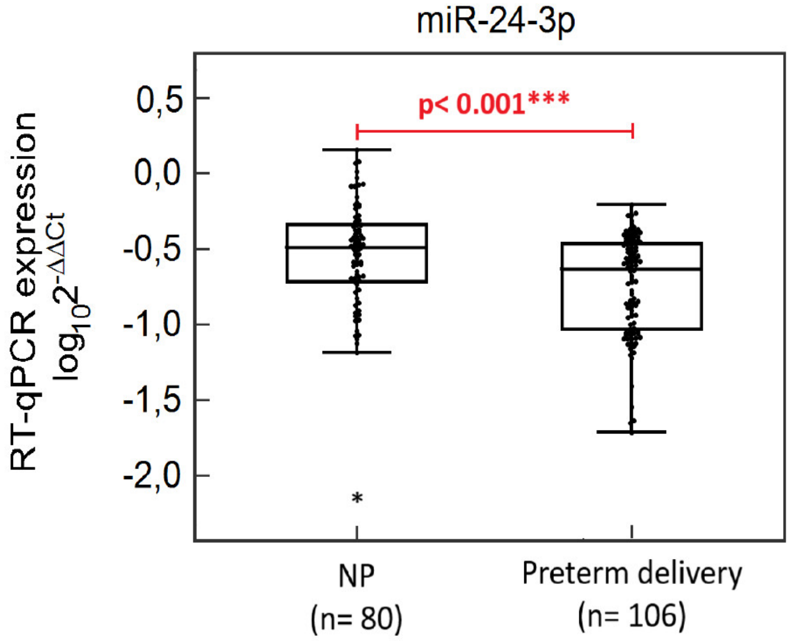

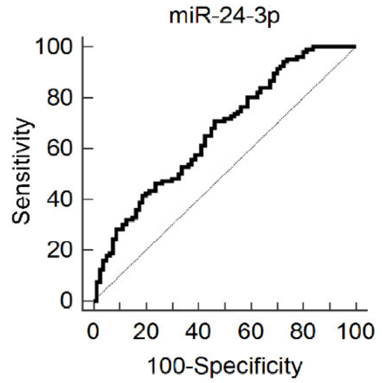

Area under the ROC curve (AUC)	0.666
Standard Error	0.0400
95% Confidence interval	0.593 to 0.733
Significance level P (Area=0.5)	<0.0001

Estimated sensitivity at fixed specificity			
Specificity	Sensitivity	95% CI	Criterion
90.00	28.30	13.21 to 41.51	≤0.107445

Criterion	Sensitivity	95% CI	Specificity	95% CI	+LR	95% CI	-LR	95% CI
≤0.319091663	70.75	61.1 - 79.2	53.75	42.2 - 65.0	1.53	1.2 - 2.0	0.54	0.4 - 0.8

E

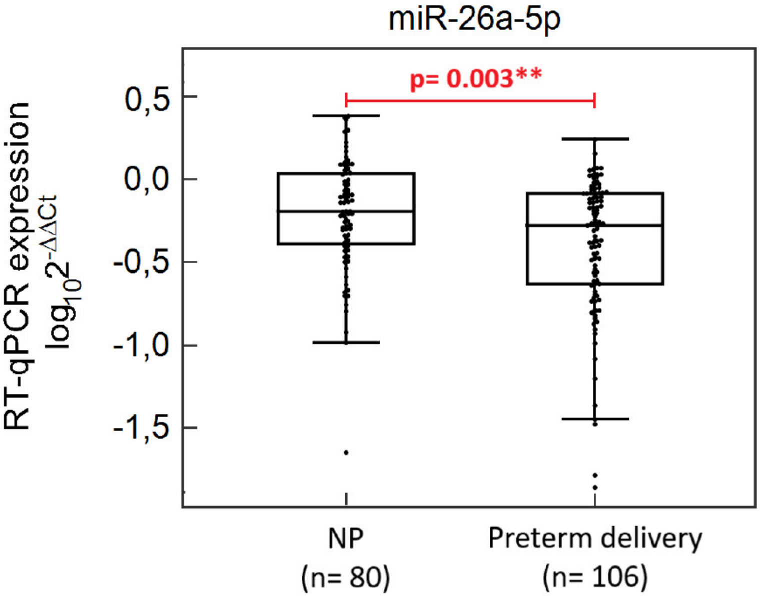

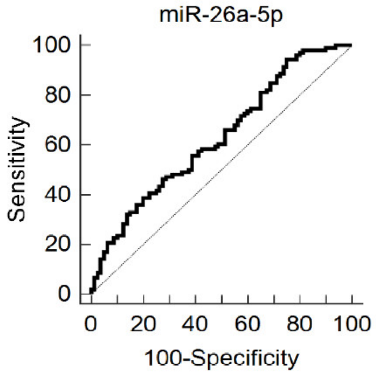

Area under the ROC curve (AUC)	0.630
Standard Error	0.0411
95% Confidence interval	0.556 to 0.699
Significance level P (Area=0.5)	0.0016

Estimated sensitivity at fixed specificity			
Specificity	Sensitivity	95% CI	Criterion
90.00	23.58	11.64 to 37.74	≤0.210206711

Criterion	Sensitivity	95% CI	Specificity	95% CI	+LR	95% CI	-LR	95% CI
≤1.076532768	94.34	88.1 - 97.9	25.00	16.0 - 35.9	1.26	1.1 - 1.4	0.23	0.10 - 0.5

F

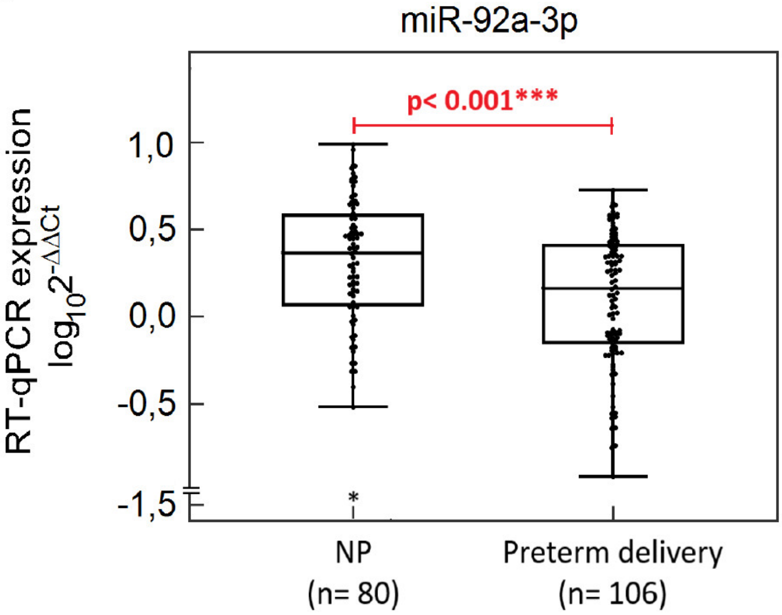

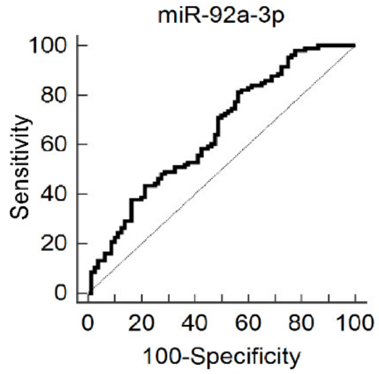

Area under the ROC curve (AUC)	0.653
Standard Error	0.0407
95% Confidence interval	0.580 to 0.721
Significance level P (Area=0.5)	0.0002

Estimated sensitivity at fixed specificity			
Specificity	Sensitivity	95% CI	Criterion
90.00	22.64	9.43 to 38.68	≤0.644813403

Criterion	Sensitivity	95% CI	Specificity	95% CI	+LR	95% CI	-LR	95% CI
≤2.777040345	81.13	72.4 - 88.1	43.75	32.7 - 55.3	1.44	1.2 - 1.8	0.43	0.3 - 0.7

G

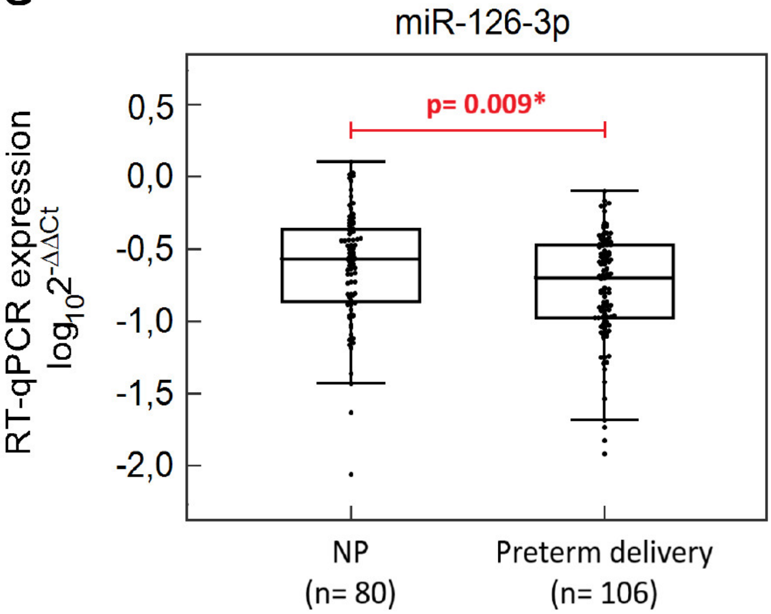

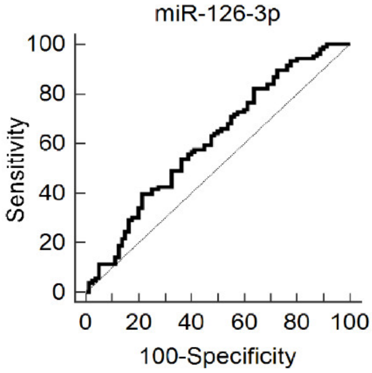

Area under the ROC curve (AUC)	0.613
Standard Error	0.0421
95% Confidence interval	0.539 to 0.683
Significance level P (Area=0.5)	0.0075

Estimated sensitivity at fixed specificity			
Specificity	Sensitivity	95% CI	Criterion
90.00	11.32	2.83 to 25.47	≤0.074792

Criterion	Sensitivity	95% CI	Specificity	95% CI	+LR	95% CI	-LR	95% CI
≤0.130922688	39.62	30.3 - 49.6	78.75	68.2 - 87.1	1.86	1.2 - 3.0	0.77	0.6 - 0.9

H

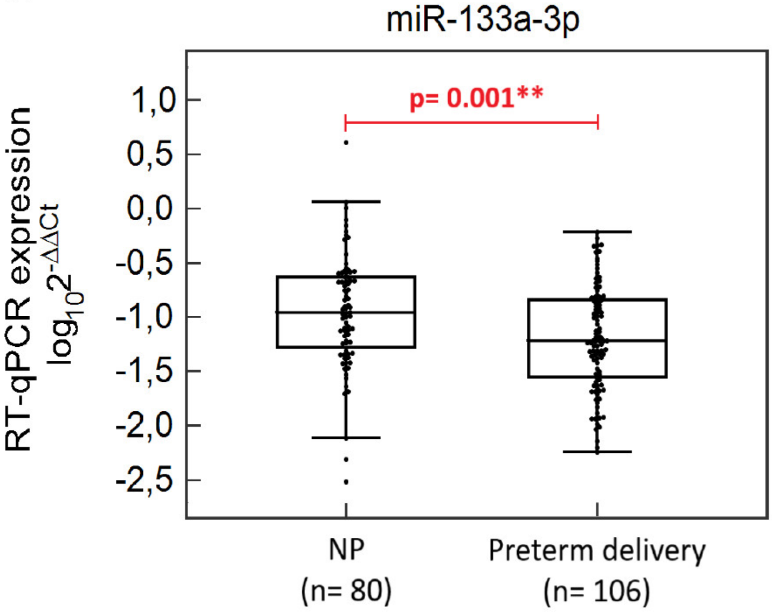

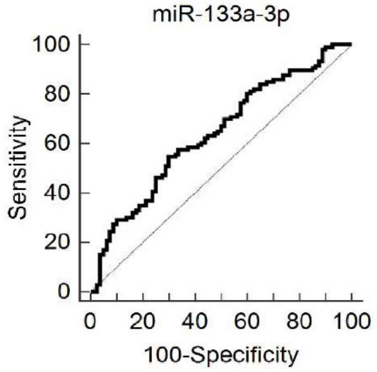

Area under the ROC curve (AUC)	0.639
Standard Error	0.0408
95% Confidence interval	0.566 to 0.708
Significance level P (Area=0.5)	0.0006

Estimated sensitivity at fixed specificity			
Specificity	Sensitivity	95% CI	Criterion
90.00	29.25	17.92 to 41.51	≤0.032746075

Criterion	Sensitivity	95% CI	Specificity	95% CI	+LR	95% CI	-LR	95% CI
≤0.065504237	54.72	44.8 - 64.4	70.00	58.7 - 79.7	1.82	1.3 - 2.7	0.65	0.5 - 0.8

I

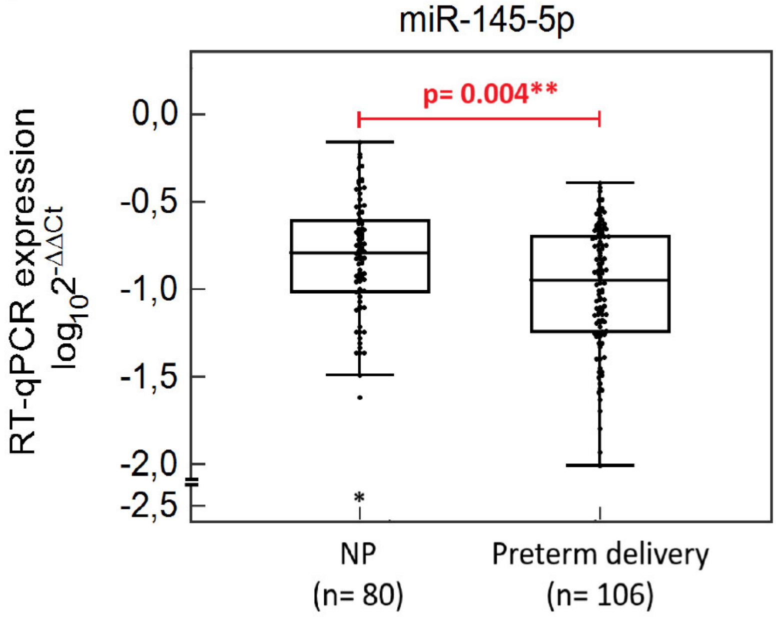

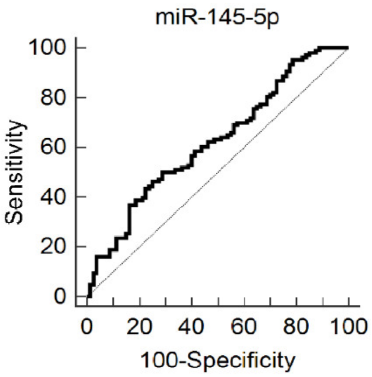

Area under the ROC curve (AUC)	0.623
Standard Error	0.0413
95% Confidence interval	0.549 to 0.693
Significance level P (Area=0.5)	0.0029

Estimated sensitivity at fixed specificity			
Specificity	Sensitivity	95% CI	Criterion
90.00	18.87	8.49 to 33.02	≤0.049521

Criterion	Sensitivity	95% CI	Specificity	95% CI	+LR	95% CI	-LR	95% CI
≤0.108623657	50.00	40.1 - 59.9	71.25	60.0 - 80.8	1.74	1.2 - 2.6	0.70	0.6 - 0.9

J

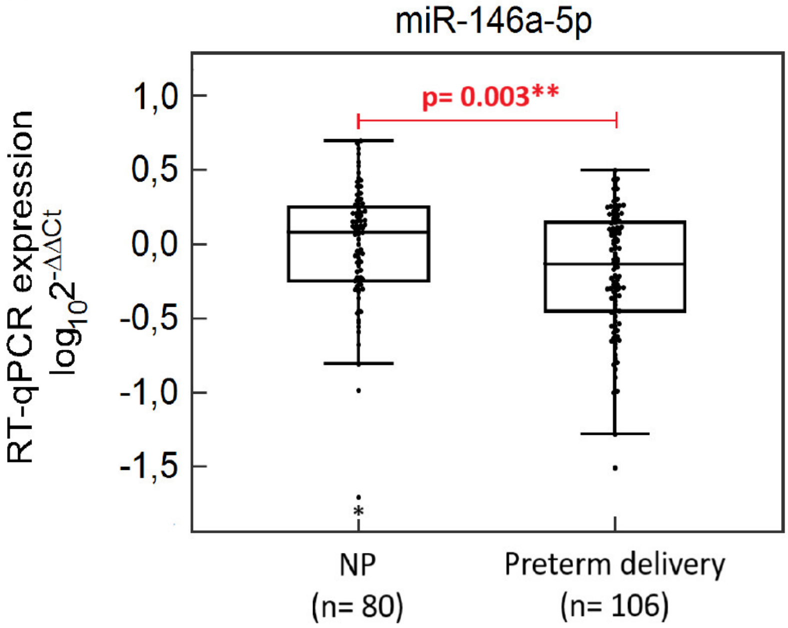

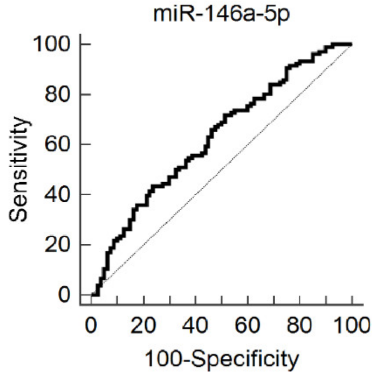

Area under the ROC curve (AUC)	0.628
Standard Error	0.0413
95% Confidence interval	0.554 to 0.698
Significance level P (Area=0.5)	0.0019

Estimated sensitivity at fixed specificity			
Specificity	Sensitivity	95% CI	Criterion
90.00	22.64	6.60 to 36.79	≤0.304521943

Criterion	Sensitivity	95% CI	Specificity	95% CI	+LR	95% CI	-LR	95% CI
≤1.279229286	71.70	62.1 - 80.0	48.75	37.4 - 60.2	1.40	1.1 - 1.8	0.58	0.4 - 0.8

K

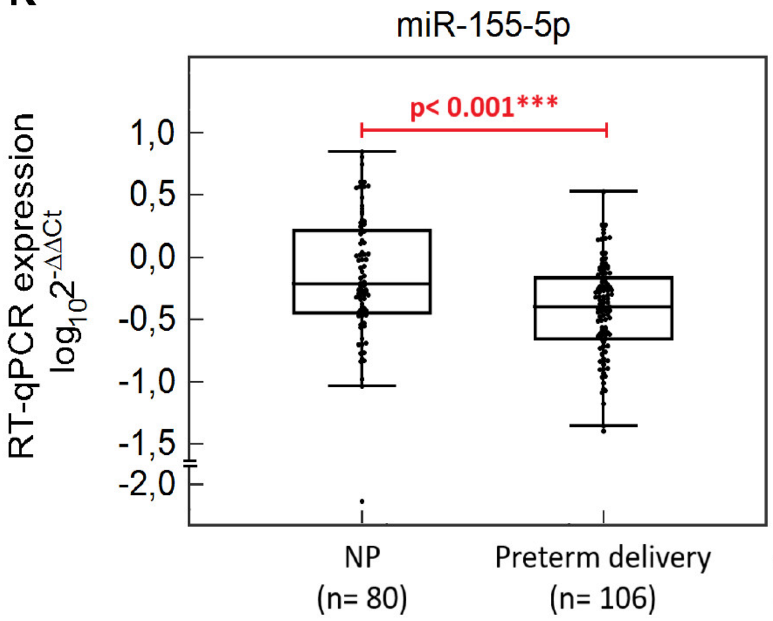

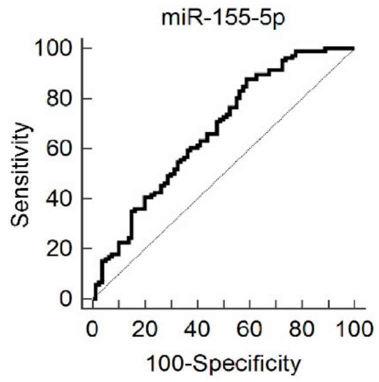

Area under the ROC curve (AUC)	0.671
Standard Error	0.0404
95% Confidence interval	0.598 to 0.738
Significance level P (Area=0.5)	<0.0001

Estimated sensitivity at fixed specificity			
Specificity	Sensitivity	95% CI	Criterion
90.00	22.64	13.21 to 45.14	≤0.194107866

Criterion	Sensitivity	95% CI	Specificity	95% CI	+LR	95% CI	-LR	95% CI
≤0.891603925	87.74	79.9 - 93.3	41.25	30.4 - 52.8	1.49	1.2 - 1.8	0.30	0.2 - 0.5

L

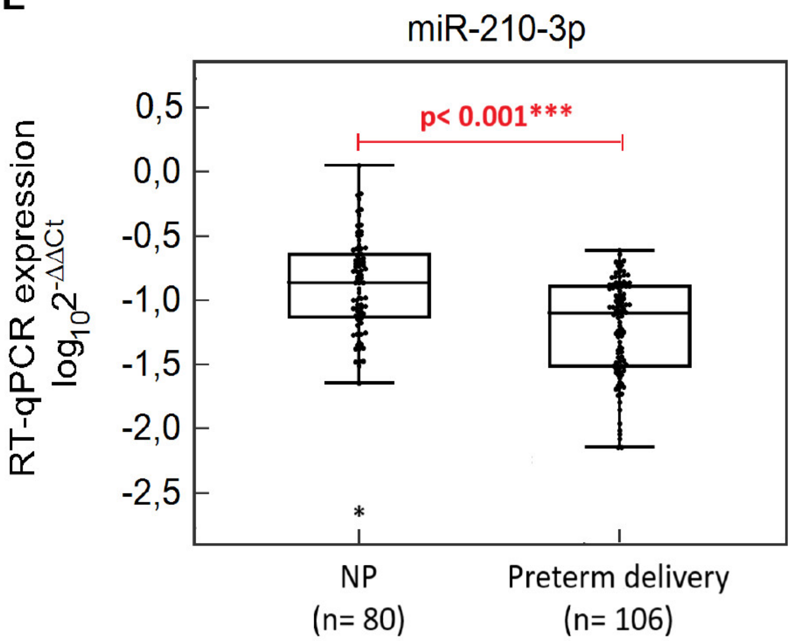

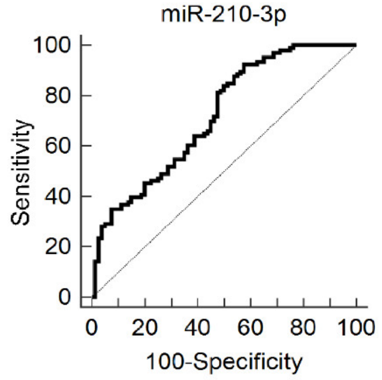

Area under the ROC curve (AUC)	0.719
Standard Error	0.0378
95% Confidence interval	0.649 to 0.782
Significance level P (Area=0.5)	<0.0001

Estimated sensitivity at fixed specificity			
Specificity	Sensitivity	95% CI	Criterion
90.00	34.91	23.69 to 46.23	≤0.041728

Criterion	Sensitivity	95% CI	Specificity	95% CI	+LR	95% CI	-LR	95% CI
≤0.167944775	92.45	85.7 - 96.7	42.50	31.5 - 54.1	1.61	1.3 - 2.0	0.18	0.09 - 0.4

M

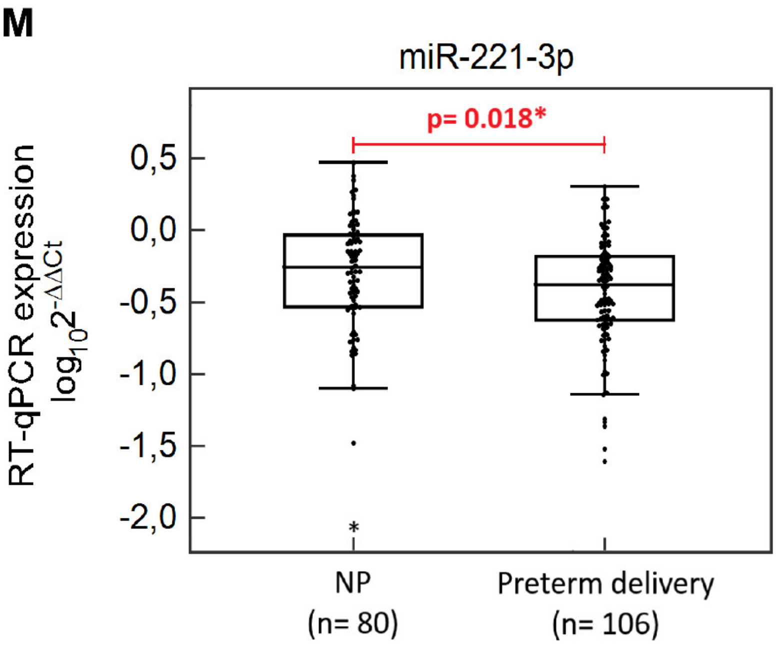

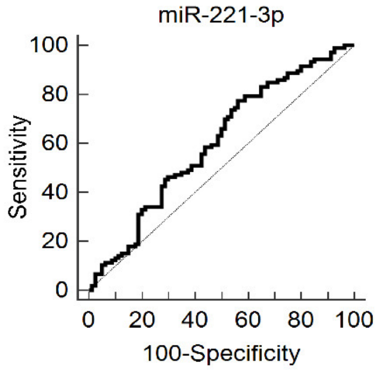

Area under the ROC curve (AUC)	0.601
Standard Error	0.0426
95% Confidence interval	0.527 to 0.672
Significance level P (Area=0.5)	0.0177

Estimated sensitivity at fixed specificity			
Specificity	Sensitivity	95% CI	Criterion
90.00	13.21	5.66 to 22.64	≤0.14758936

Criterion	Sensitivity	95% CI	Specificity	95% CI	+LR	95% CI	-LR	95% CI
≤0.670487829	77.36	68.2 - 84.9	43.75	32.7 - 55.3	1.38	1.1 - 1.7	0.52	0.3 - 0.8

N

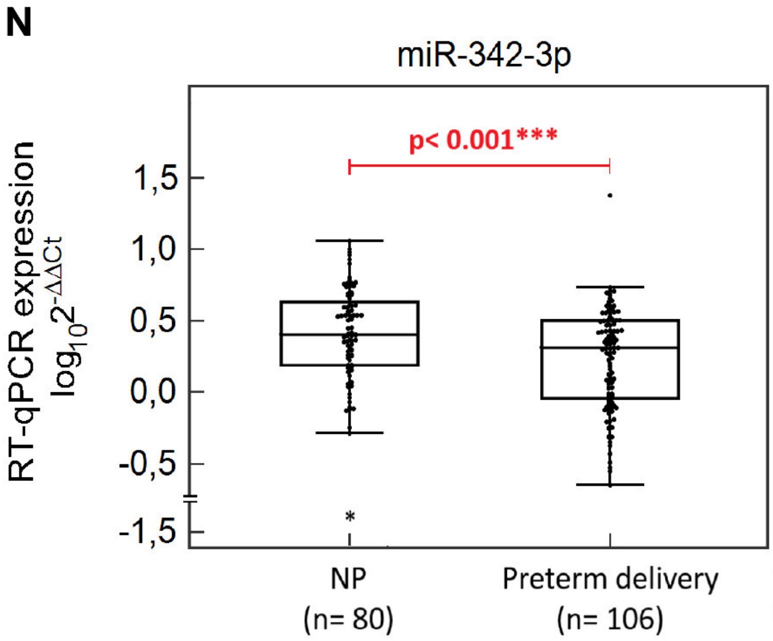

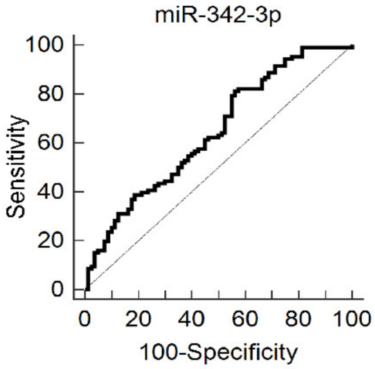

Area under the ROC curve (AUC)	0.644
Standard Error	0.0409
95% Confidence interval	0.570 to 0.712
Significance level P (Area=0.5)	0.0004

Estimated sensitivity at fixed specificity			
Specificity	Sensitivity	95% CI	Criterion
90.00	25.47	10.38 to 38.68	≤0.903942535

Criterion	Sensitivity	95% CI	Specificity	95% CI	+LR	95% CI	-LR	95% CI
≤3.281807058	81.13	72.4 - 88.1	43.75	32.7 - 55.3	1.44	1.2 - 1.8	0.43	0.3 - 0.7

Supplementary Figure S1: Gene expression of cardiovascular disease associated microRNAs in peripheral blood leukocytes in early stages of gestation – comparison between NP and preterm delivery – statistical significant data after Benjamini-Hochberg correction (results after the Benjamini-Hochberg correction are marked by * for α=0.05, ** for α=0.01, and *** for α=0.001). NP, normal pregnancies.
