# Supplementary material for: First Trimester Prediction of Preterm Delivery in the Absence of Other Pregnancy-Related Complications Using Cardiovascular-Disease Associated MicroRNA Biomarkers
Source: Int J Mol Sci. 2022 Apr 1;23(7):3951. doi: 10.3390/ijms23073951 (PMC8999783; doi:10.3390/ijms23073951)
Supplement: Supplementary file 1 [file ijms-23-03951-s001.zip › Supplementary Figure S2.pdf]

Supplementary Figure S2.

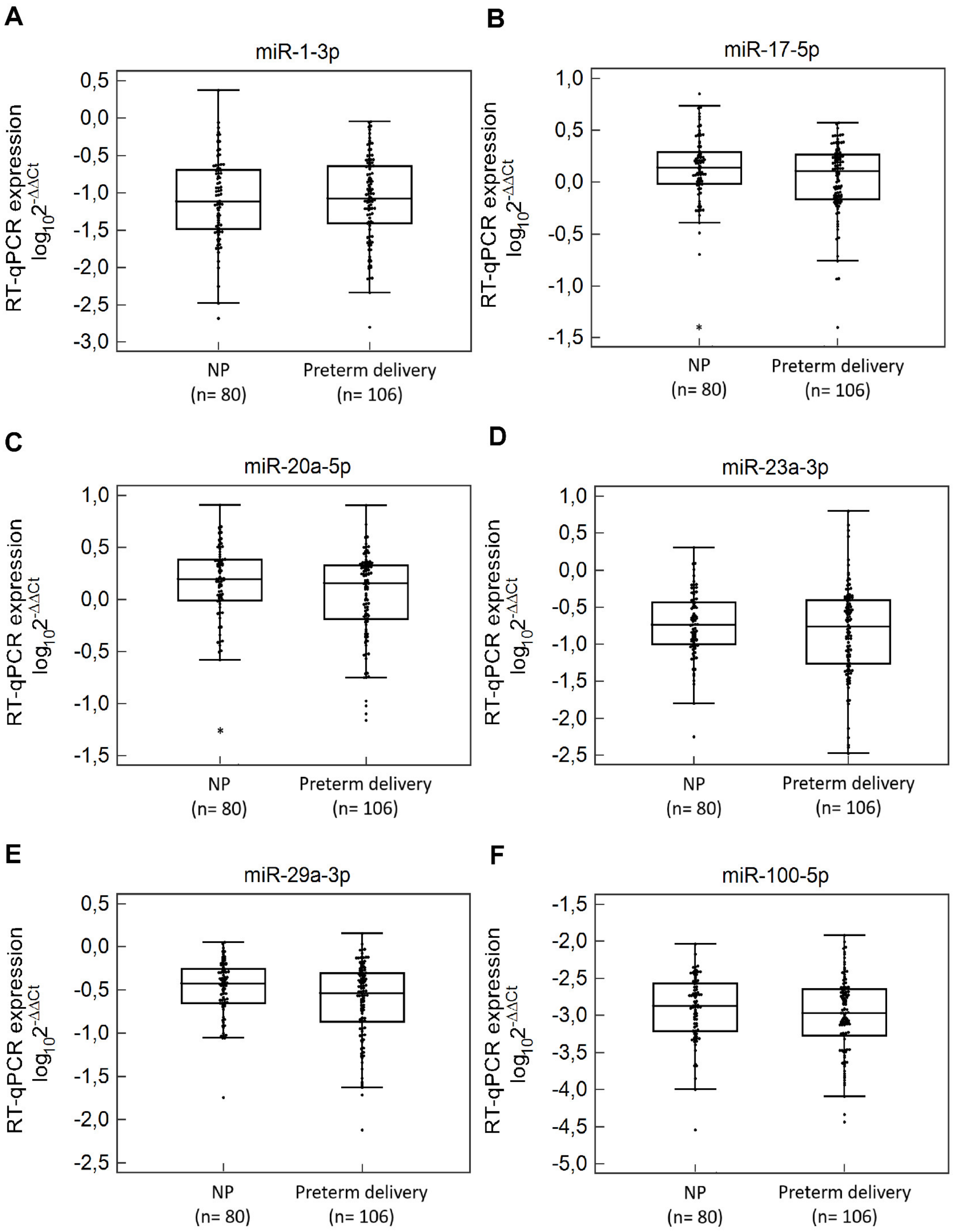

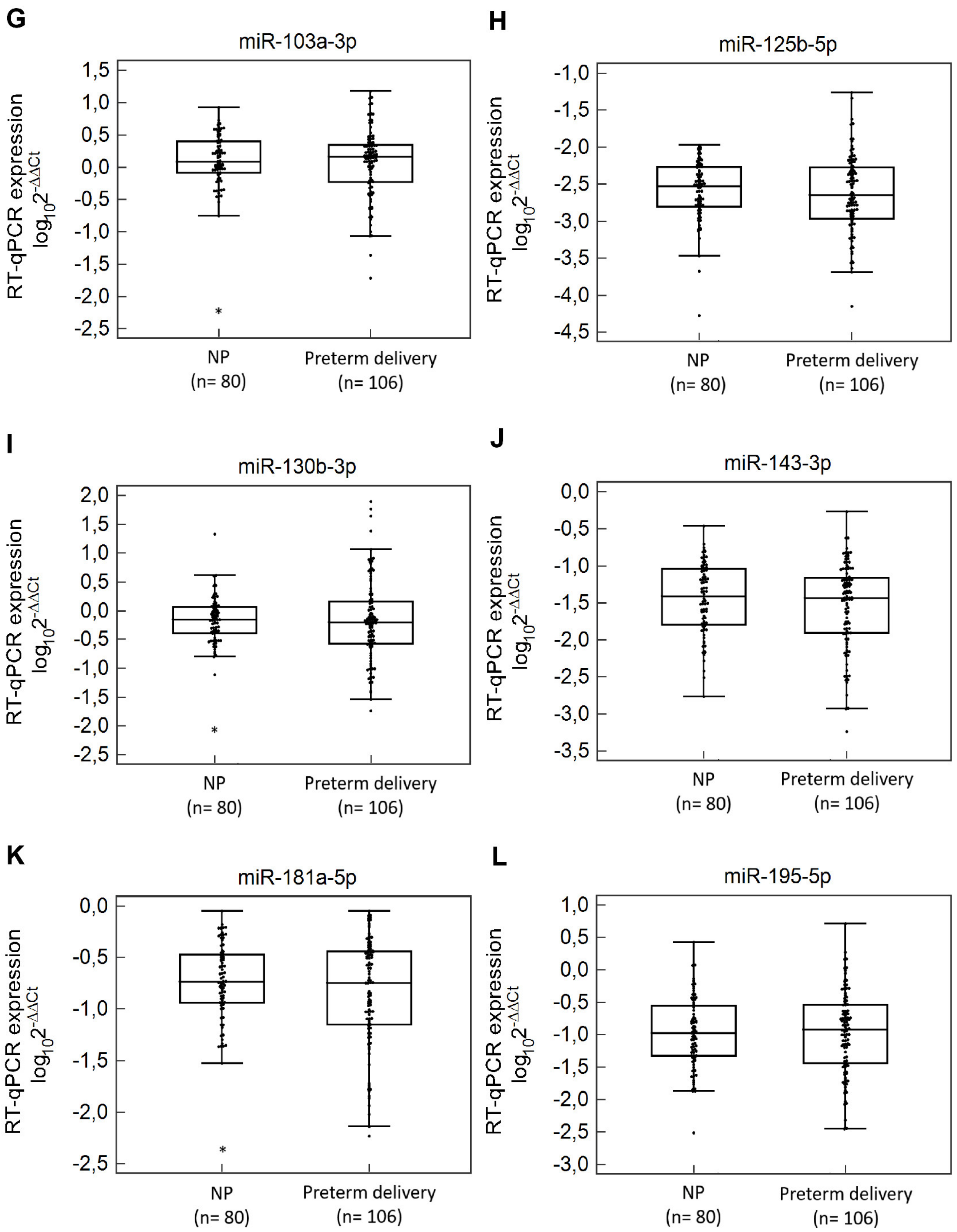

G
miR-103a-3p
RT-qPCR expression
log10 2-ΔΔCt
1,5
1,0
0,5
0,0
-0,5
-1,0
-1,5
-2,0
-2,5
NP
(n= 80)
Preterm delivery
(n= 106)
H
miR-125b-5p
RT-qPCR expression
log10 2-ΔΔCt
-1,0
-1,5
-2,0
-2,5
-3,0
-3,5
-4,0
-4,5
NP
(n= 80)
Preterm delivery
(n= 106)
I
miR-130b-3p
RT-qPCR expression
log10 2-ΔΔCt
2,0
1,5
1,0
0,5
0,0
-0,5
-1,0
-1,5
-2,0
-2,5
NP
(n= 80)
Preterm delivery
(n= 106)
J
miR-143-3p
RT-qPCR expression
log10 2-ΔΔCt
0,0
-0,5
-1,0
-1,5
-2,0
-2,5
-3,0
-3,5
NP
(n= 80)
Preterm delivery
(n= 106)
K
miR-181a-5p
RT-qPCR expression
log10 2-ΔΔCt
0,0
-0,5
-1,0
-1,5
-2,0
-2,5
NP
(n= 80)
Preterm delivery
(n= 106)
L
miR-195-5p
RT-qPCR expression
log10 2-ΔΔCt
1,0
0,5
0,0
-0,5
-1,0
-1,5
-2,0
-2,5
-3,0
NP
(n= 80)
Preterm delivery
(n= 106)

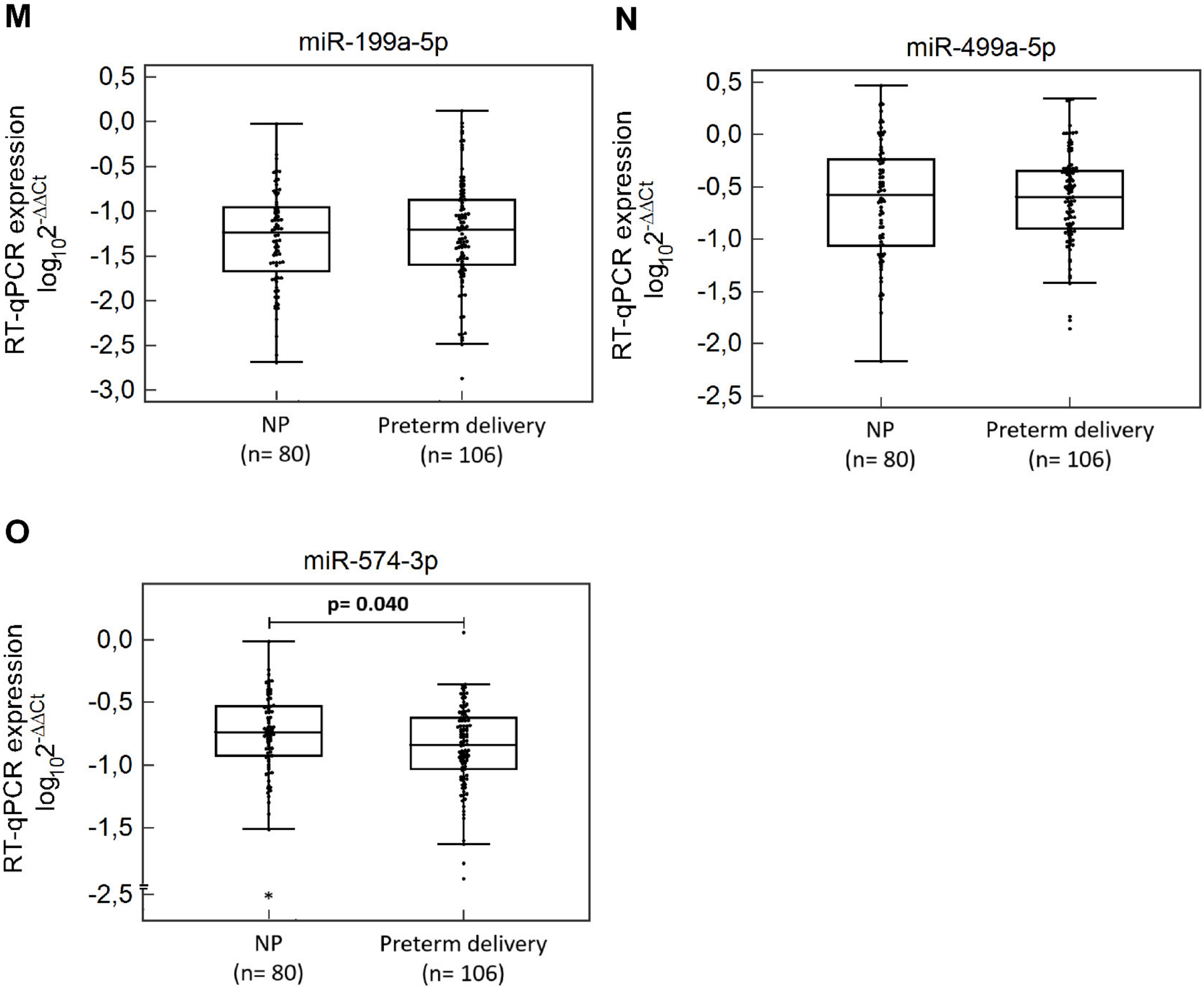

Supplementary Figure S2. Gene expression of cardiovascular disease associated microRNAs in peripheral blood leukocytes in early stages of gestation – comparison between NP and preterm delivery – statistical non-significant data after Benjamini-Hochberg correction. NP, normal pregnancies.
