# Supplementary material for: First Trimester Prediction of Preterm Delivery in the Absence of Other Pregnancy-Related Complications Using Cardiovascular-Disease Associated MicroRNA Biomarkers
Source: Int J Mol Sci. 2022 Apr 1;23(7):3951. doi: 10.3390/ijms23073951 (PMC8999783; doi:10.3390/ijms23073951)
Supplement: Supplementary file 1 [file ijms-23-03951-s001.zip › Supplementary Figure S3.pdf]

Supplementary Figure S3.

A

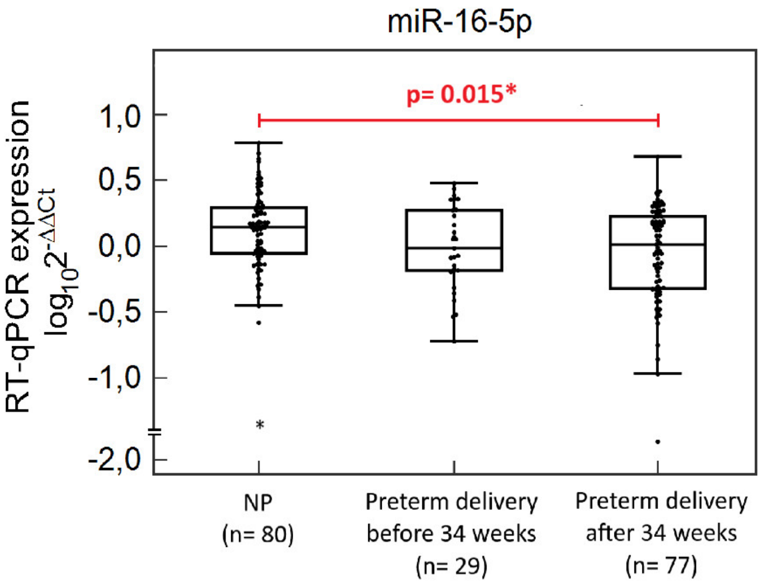

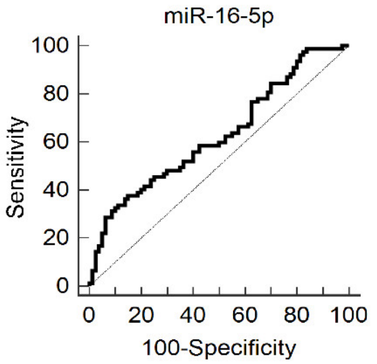

Area under the ROC curve (AUC)	0.627
Standard Error	0.0446
95% Confidence interval	0.546 to 0.703
Significance level P (Area=0.5)	0.0045

Estimated sensitivity at fixed specificity			
Specificity	Sensitivity	95% CI	Criterion
90.00	32.47	16.88 to 45.45	≤0.597445814

Criterion	Sensitivity	95% CI	Specificity	95% CI	+LR	95% CI	-LR	95% CI
≤0.721850094	37.66	26.9 - 49.4	85.00	75.3 - 92.0	2.51	1.4 - 4.6	0.73	0.6 - 0.9

B

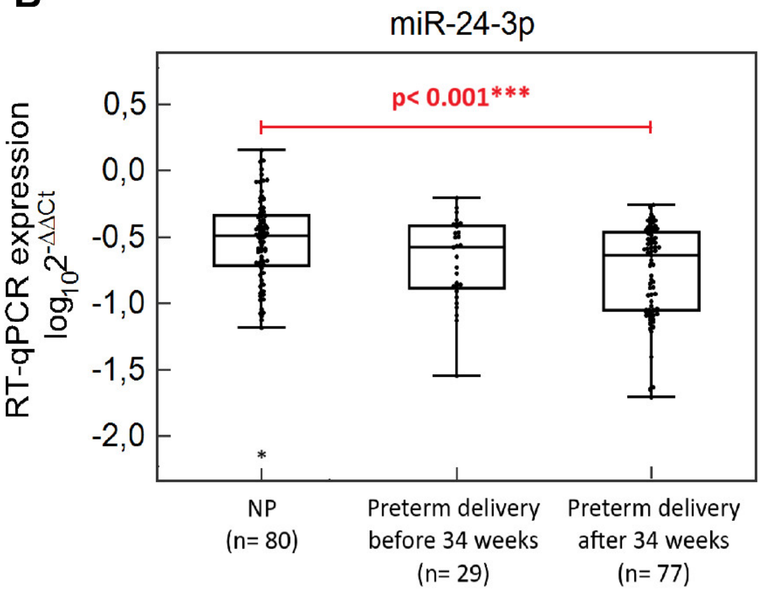

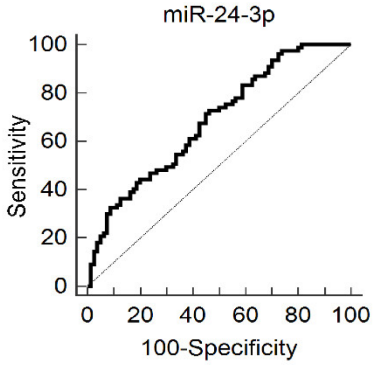

Area under the ROC curve (AUC)	0.684
Standard Error	0.0420
95% Confidence interval	0.605 to 0.756
Significance level P (Area=0.5)	<0.0001

Estimated sensitivity at fixed specificity			
Specificity	Sensitivity	95% CI	Criterion
90.00	32.47	16.88 to 46.75	≤0.107445

Criterion	Sensitivity	95% CI	Specificity	95% CI	+LR	95% CI	-LR	95% CI
≤0.309313239	72.73	61.4 - 82.3	53.75	42.2 - 65.0	1.57	1.2 - 2.1	0.51	0.3 - 0.8

C

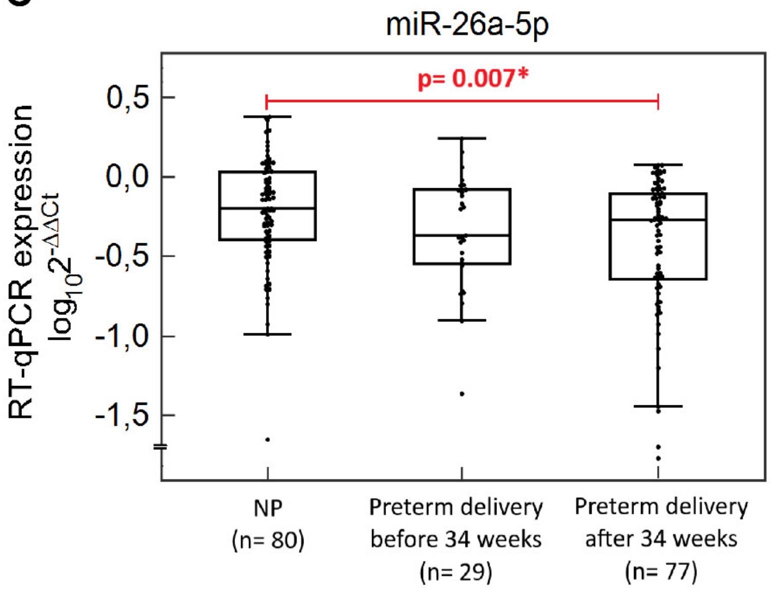

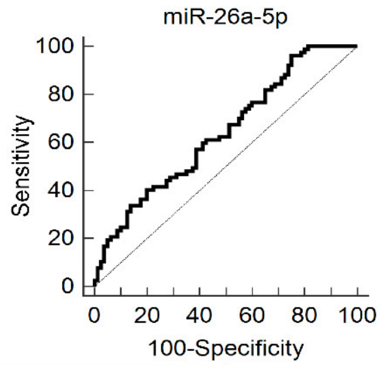

Area under the ROC curve (AUC)	0.640
Standard Error	0.0438
95% Confidence interval	0.559 to 0.715
Significance level P (Area=0.5)	0.0014

Estimated sensitivity at fixed specificity			
Specificity	Sensitivity	95% CI	Criterion
90.00	24.68	12.99 to 40.26	≤0.210206711

Criterion	Sensitivity	95% CI	Specificity	95% CI	+LR	95% CI	-LR	95% CI
≤1.076532768	96.10	89.0 - 99.2	25.00	16.0 - 35.9	1.28	1.1 - 1.5	0.16	0.05 - 0.5

D

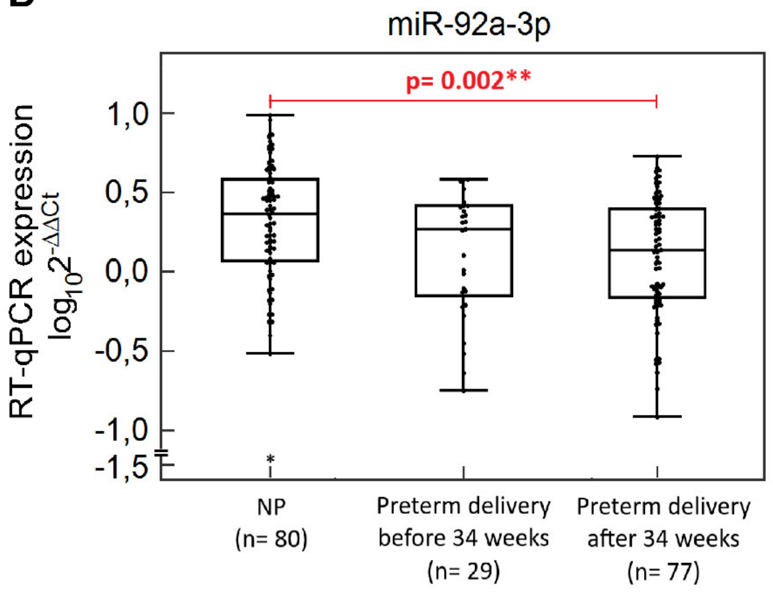

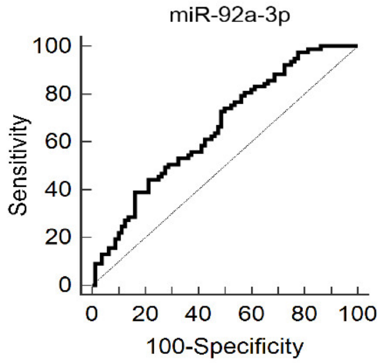

Area under the ROC curve (AUC)	0.658
Standard Error	0.0432
95% Confidence interval	0.578 to 0.732
Significance level P (Area=0.5)	0.0003

Estimated sensitivity at fixed specificity			
Specificity	Sensitivity	95% CI	Criterion
90.00	22.08	9.09 to 42.86	≤0.644813403

Criterion	Sensitivity	95% CI	Specificity	95% CI	+LR	95% CI	-LR	95% CI
≤2.351230095	74.03	62.8 - 83.4	50.00	38.6 - 61.4	1.48	1.1 - 1.9	0.52	0.3 - 0.8

E

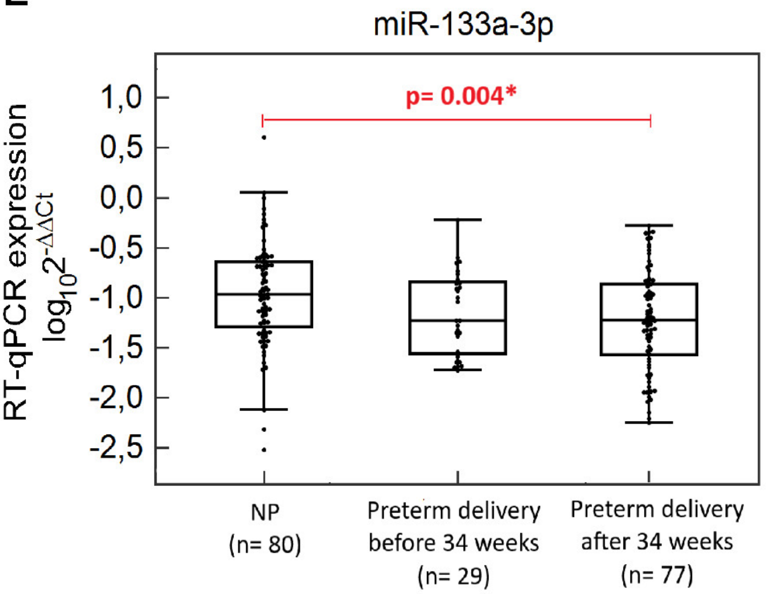

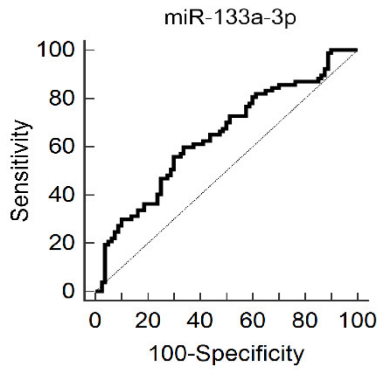

Area under the ROC curve (AUC)	0.644
Standard Error	0.0441
95% Confidence interval	0.564 to 0.719
Significance level P (Area=0.5)	0.0011

Estimated sensitivity at fixed specificity			
Specificity	Sensitivity	95% CI	Criterion
90.00	29.87	18.18 to 44.33	≤0.032746075

Criterion	Sensitivity	95% CI	Specificity	95% CI	+LR	95% CI	-LR	95% CI
≤0.073441271	59.74	47.9 - 70.8	66.25	54.8 - 76.4	1.77	1.2 - 2.5	0.61	0.4 - 0.8

F

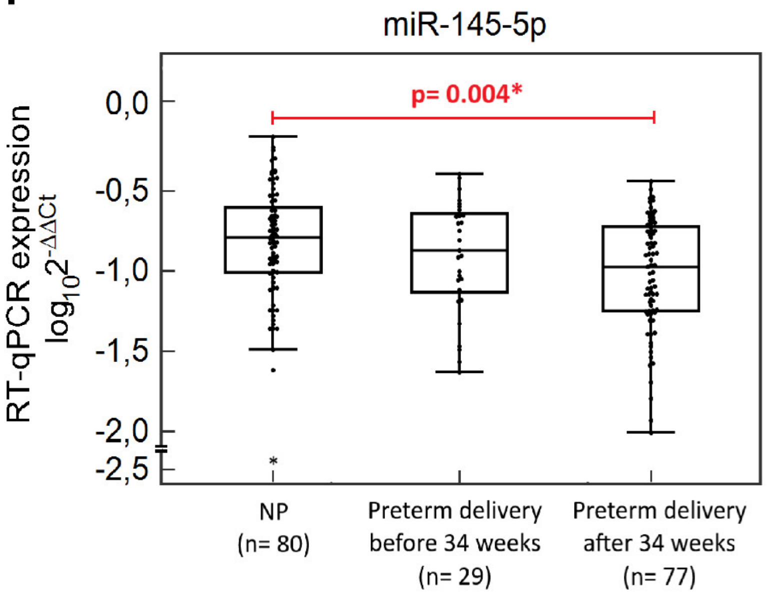

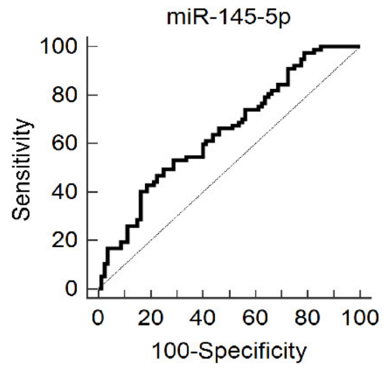

Area under the ROC curve (AUC)	0.649
Standard Error	0.0436
95% Confidence interval	0.569 to 0.723
Significance level P (Area=0.5)	0.0007

Estimated sensitivity at fixed specificity			
Specificity	Sensitivity	95% CI	Criterion
90.00	19.48	7.79 to 36.36	≤0.049521

Criterion	Sensitivity	95% CI	Specificity	95% CI	+LR	95% CI	-LR	95% CI
≤0.108623657	53.25	41.5 - 64.7	71.25	60.0 - 80.8	1.85	1.2 - 2.8	0.66	0.5 - 0.9

G

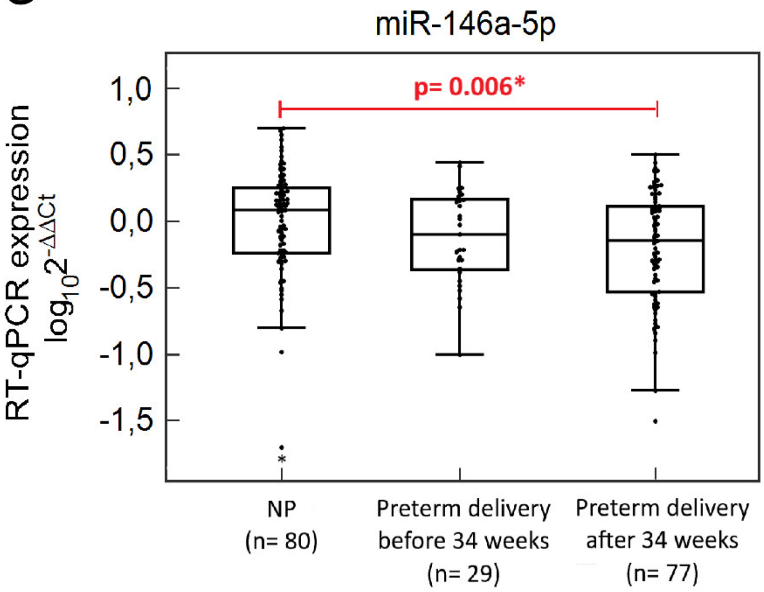

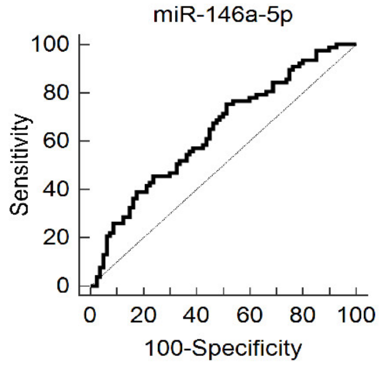

Area under the ROC curve (AUC)	0.641
Standard Error [a]	0.0440
95% Confidence interval [b]	0.561 to 0.716
z statistic	3.206
Significance level P (Area=0.5)	0.0013

Estimated sensitivity at fixed specificity			
Specificity	Sensitivity	95% CI [a]	Criterion
90.00	25.97	9.09 to 42.27	≤0.301884

Criterion	Sensitivity	95% CI	Specificity	95% CI	+LR	95% CI	-LR	95% CI
≤1.279229286	75.32	64.2 - 84.4	48.75	37.4 - 60.2	1.47	1.1 - 1.9	0.51	0.3 - 0.8

H

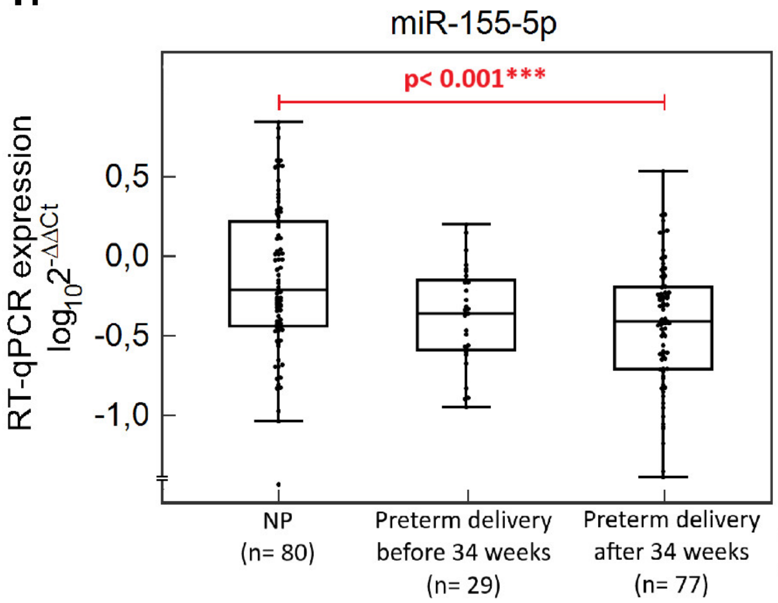

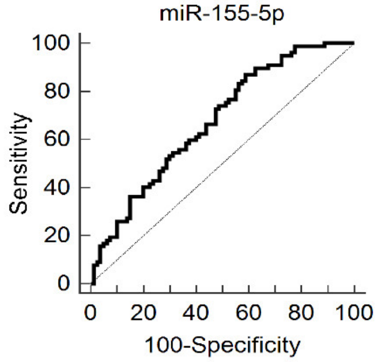

Area under the ROC curve (AUC)	0.675
Standard Error	0.0425
95% Confidence interval	0.595 to 0.747
Significance level P (Area=0.5)	<0.0001

Estimated sensitivity at fixed specificity			
Specificity	Sensitivity	95% CI	Criterion
90.00	25.97	12.99 to 48.05	≤0.194107866

Criterion	Sensitivity	95% CI	Specificity	95% CI	+LR	95% CI	-LR	95% CI
≤0.891603925	87.01	77.4 - 93.6	41.25	30.4 - 52.8	1.48	1.2 - 1.8	0.31	0.2 - 0.6

I

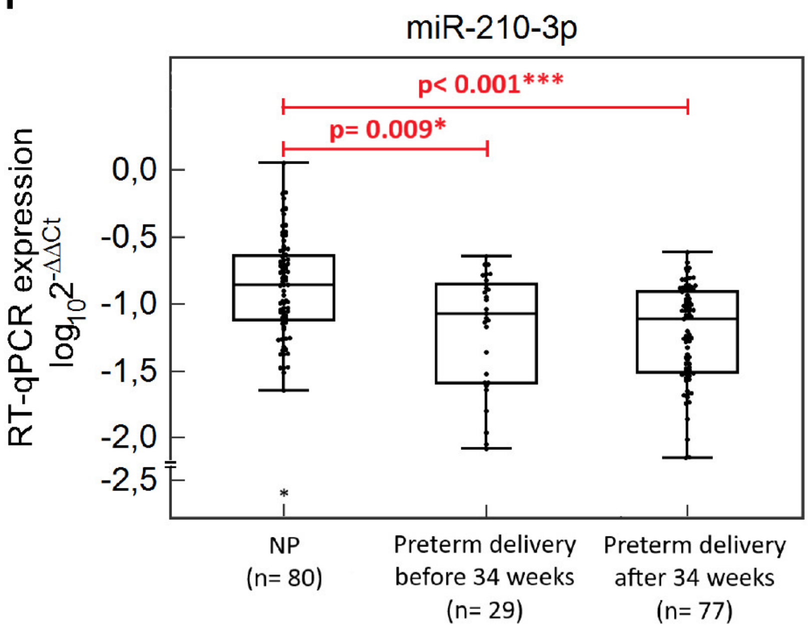

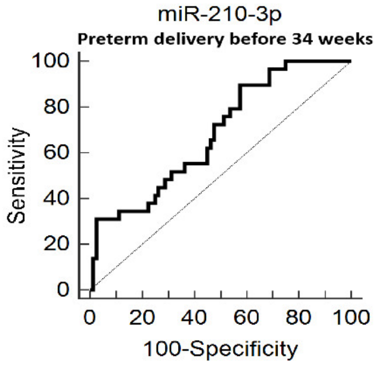

Area under the ROC curve (AUC)	0.683
Standard Error	0.0558
95% Confidence interval	0.587 to 0.769
Significance level P (Area=0.5)	0.0010

Estimated sensitivity at fixed specificity			
Specificity	Sensitivity	95% CI	Criterion
90.00	31.03	13.79 to 48.28	≤0.041728

Criterion	Sensitivity	95% CI	Specificity	95% CI	+LR	95% CI	-LR	95% CI
≤0.167944775	89.66	72.6 - 97.8	42.50	31.5 - 54.1	1.56	1.2 - 2.0	0.24	0.08 - 0.7

J

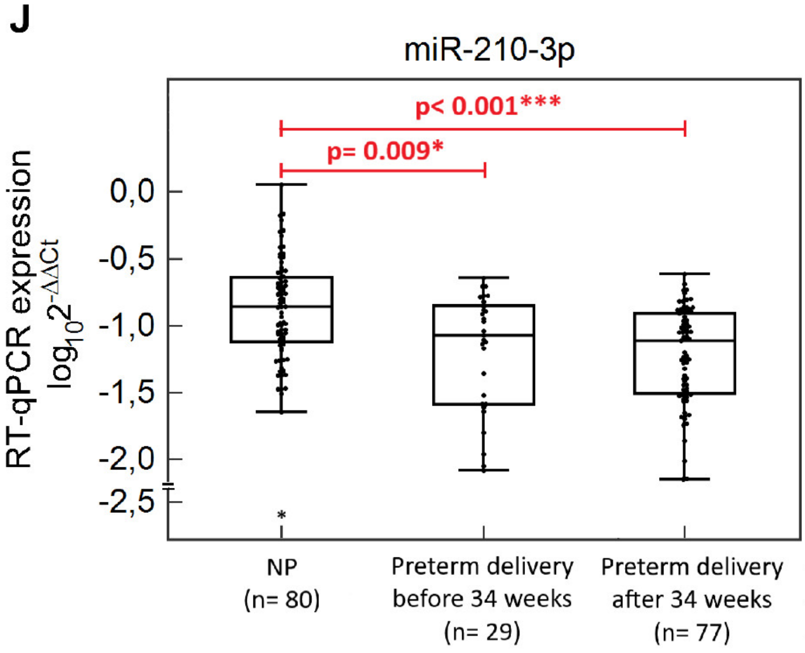

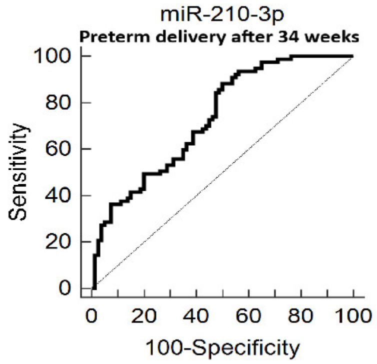

Area under the ROC curve (AUC)	0.733
Standard Error	0.0394
95% Confidence interval	0.656 to 0.800
Significance level P (Area=0.5)	<0.0001

Estimated sensitivity at fixed specificity			
Specificity	Sensitivity	95% CI	Criterion
90.00	36.36	22.08 to 49.35	≤0.041728

Criterion	Sensitivity	95% CI	Specificity	95% CI	+LR	95% CI	-LR	95% CI
≤0.13768547	88.31	79.0 - 94.5	50.00	38.6 - 61.4	1.77	1.4 - 2.2	0.23	0.1 - 0.4

K

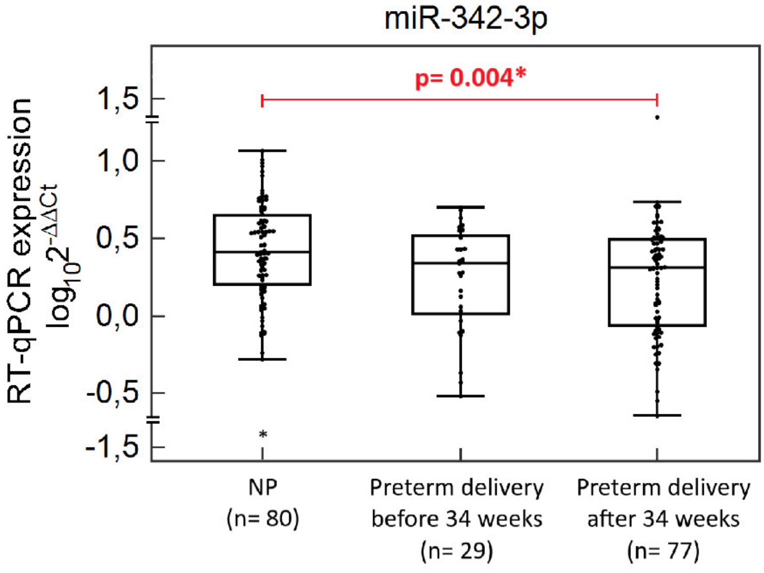

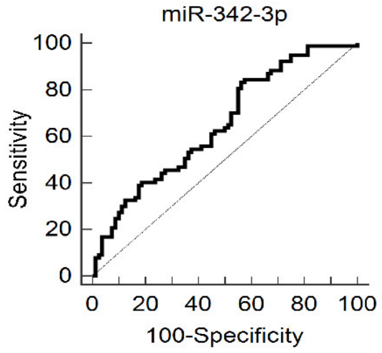

Area under the ROC curve (AUC)	0.649
Standard Error	0.0436
95% Confidence interval	0.569 to 0.724
Significance level P (Area=0.5)	0.0006

Estimated sensitivity at fixed specificity			
Specificity	Sensitivity	95% CI	Criterion
90.00	27.27	11.99 to 44.16	≤0.903942535

Criterion	Sensitivity	95% CI	Specificity	95% CI	+LR	95% CI	-LR	95% CI
≤3.407103378	84.42	74.4 - 91.7	42.50	31.5 - 54.1	1.47	1.2 - 1.8	0.37	0.2 - 0.7

Supplementary Figure S3: Gene expression of cardiovascular disease associated microRNAs in peripheral blood leukocytes in early stages of gestation – comparison between NP and preterm delivery before and after 34 weeks of gestation – statistical significant data after Benjamini-Hochberg correction (results after the Benjamini-Hochberg correction are marked by * for α=0.05, ** for α=0.01, and *** for α=0.001). NP, normal pregnancies.
