# Supplementary material for: First Trimester Prediction of Preterm Delivery in the Absence of Other Pregnancy-Related Complications Using Cardiovascular-Disease Associated MicroRNA Biomarkers
Source: Int J Mol Sci. 2022 Apr 1;23(7):3951. doi: 10.3390/ijms23073951 (PMC8999783; doi:10.3390/ijms23073951)
Supplement: Supplementary file 1 [file ijms-23-03951-s001.zip › Supplementary Figure S5.pdf]

Supplementary Figure S5.

A

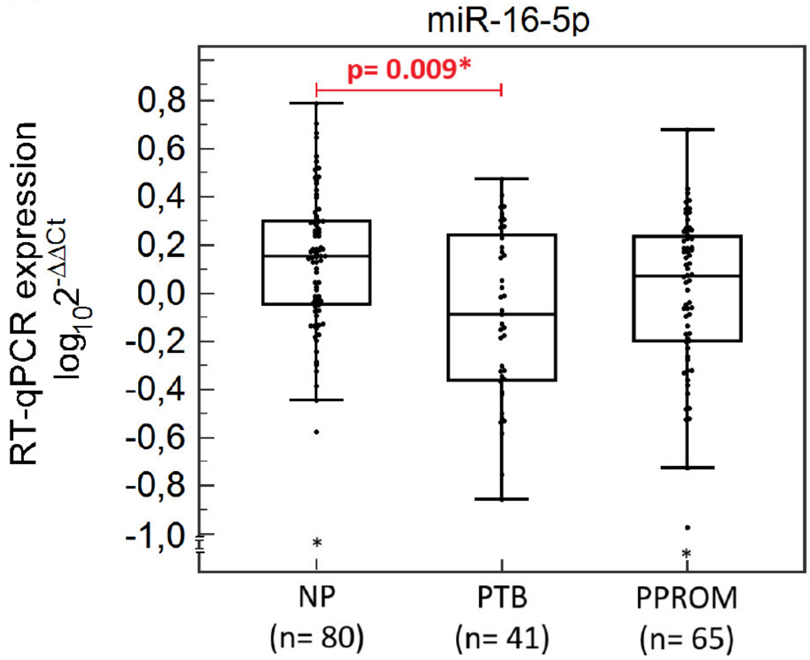

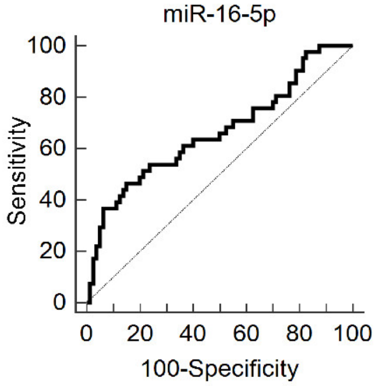

Area under the ROC curve (AUC)	0.663
Standard Error	0.0551
95% Confidence interval	0.571 to 0.746
Significance level P (Area=0.5)	0.0032

Estimated sensitivity at fixed specificity			
Specificity	Sensitivity	95% CI	Criterion
90.00	36.59	17.07 to 51.22	≤0.568076

Criterion	Sensitivity	95% CI	Specificity	95% CI	+LR	95% CI	-LR	95% CI
≤0.721850094	46.34	30.7 - 62.6	85.00	75.3 - 92.0	3.09	1.7 - 5.7	0.63	0.5 - 0.9

B

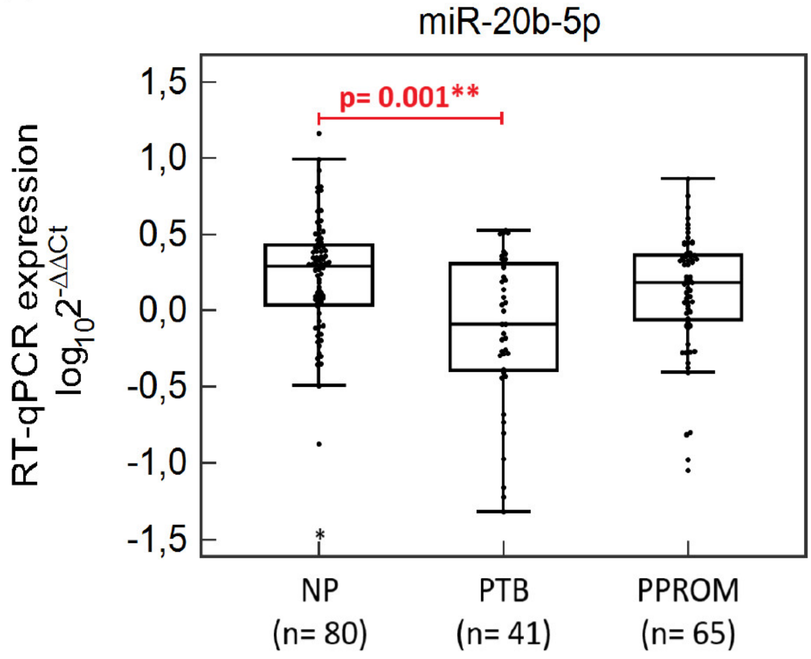

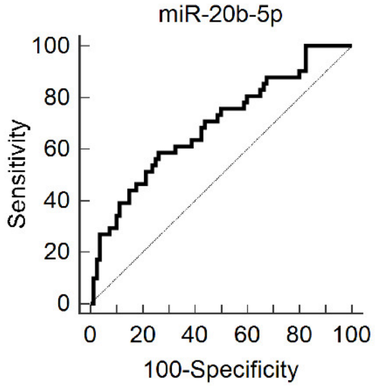

Area under the ROC curve (AUC)	0.691
Standard Error	0.0518
95% Confidence interval	0.600 to 0.771
Significance level P (Area=0.5)	0.0002

Estimated sensitivity at fixed specificity			
Specificity	Sensitivity	95% CI	Criterion
90.00	34.15	17.07 to 51.22	≤0.523427

Criterion	Sensitivity	95% CI	Specificity	95% CI	+LR	95% CI	-LR	95% CI
≤1.131364979	58.54	42.1 - 73.7	73.75	62.7 - 83.0	2.23	1.4 - 3.5	0.56	0.4 - 0.8

C

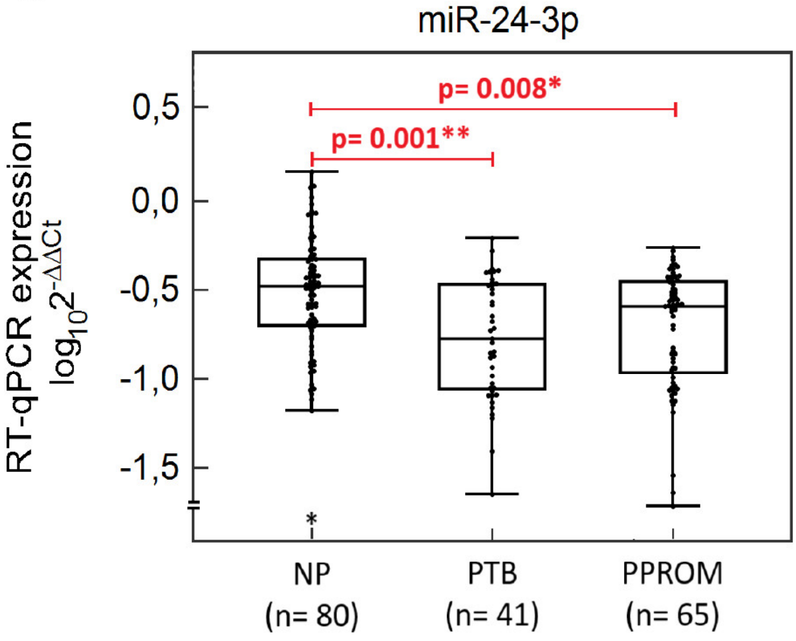

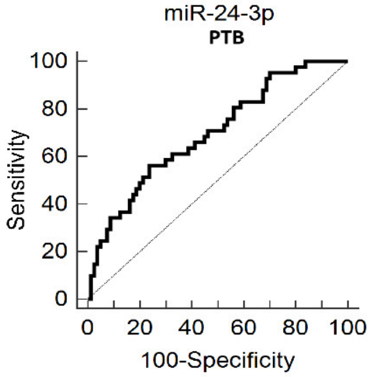

Area under the ROC curve (AUC)	0.696
Standard Error	0.0501
95% Confidence interval	0.606 to 0.776
Significance level P (Area=0.5)	0.0001

Estimated sensitivity at fixed specificity			
Specificity	Sensitivity	95% CI	Criterion
90.00	34.15	17.07 to 51.22	≤0.107445

Criterion	Sensitivity	95% CI	Specificity	95% CI	+LR	95% CI	-LR	95% CI
≤0.193516	56.10	39.7 - 71.5	75.00	64.1 - 84.0	2.24	1.4 - 3.6	0.59	0.4 - 0.8

D

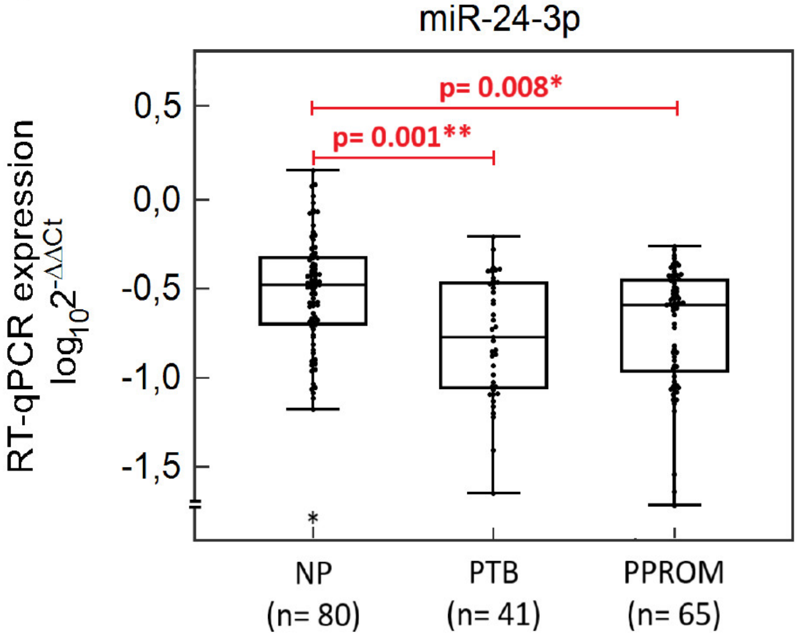

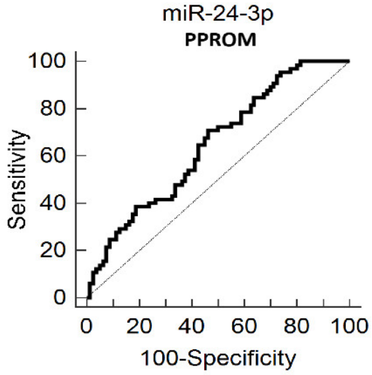

Area under the ROC curve (AUC)	0.647
Standard Error	0.0453
95% Confidence interval	0.564 to 0.725
Significance level P (Area=0.5)	0.0012

Estimated sensitivity at fixed specificity			
Specificity	Sensitivity	95% CI	Criterion
90.00	24.62	9.81 to 40.00	≤0.107445

Criterion	Sensitivity	95% CI	Specificity	95% CI	+LR	95% CI	-LR	95% CI
≤0.316368821	70.77	58.2 - 81.4	53.75	42.2 - 65.0	1.53	1.2 - 2.0	0.54	0.4 - 0.8

E

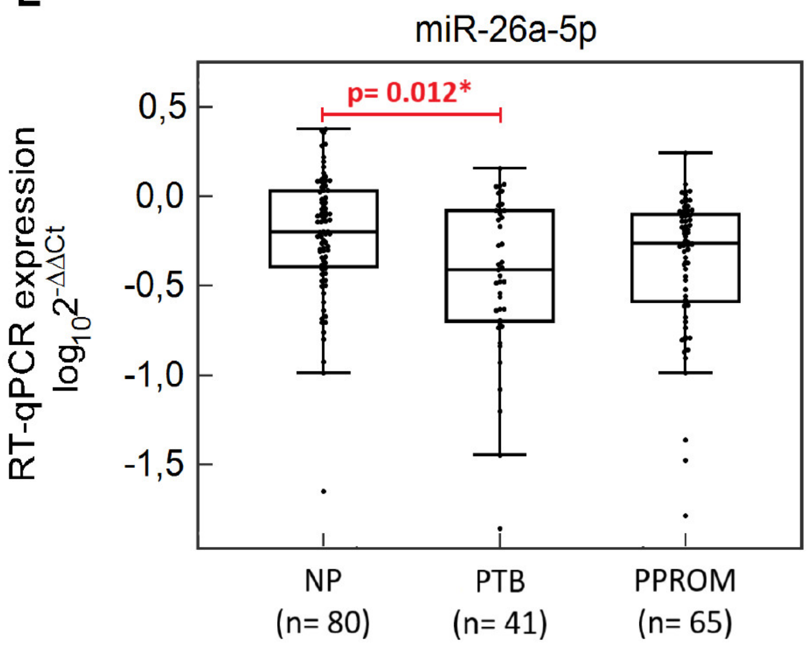

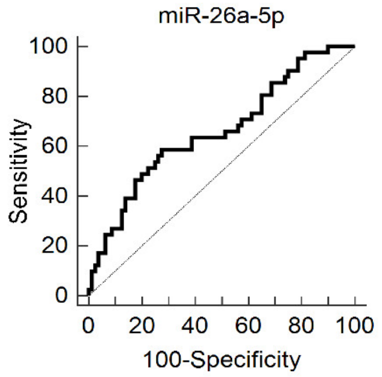

Area under the ROC curve (AUC)	0.660
Standard Error	0.0536
95% Confidence interval	0.568 to 0.744
Significance level P (Area=0.5)	0.0028

Estimated sensitivity at fixed specificity			
Specificity	Sensitivity	95% CI	Criterion
90.00	26.83	9.76 to 46.34	≤0.206064

Criterion	Sensitivity	95% CI	Specificity	95% CI	+LR	95% CI	-LR	95% CI
≤0.427558781	58.54	42.1 - 73.7	72.50	61.4 - 81.9	2.13	1.4 - 3.3	0.57	0.4 - 0.8

F

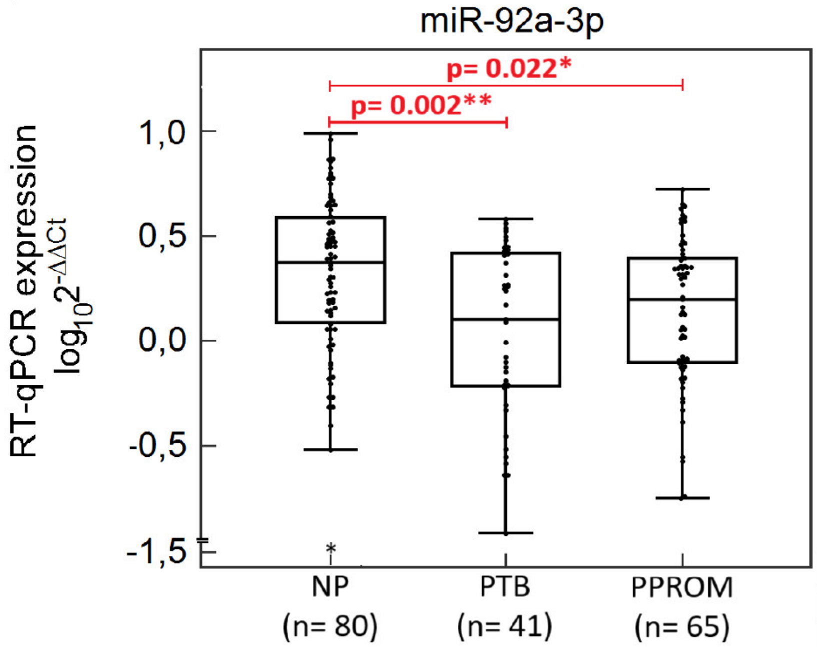

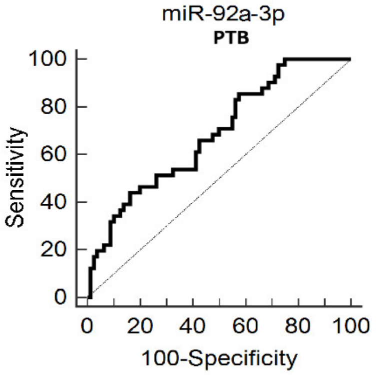

Area under the ROC curve (AUC)	0.683
Standard Error	0.0501
95% Confidence interval	0.592 to 0.765
Significance level P (Area=0.5)	0.0003

Estimated sensitivity at fixed specificity			
Specificity	Sensitivity	95% CI	Criterion
90.00	34.15	14.63 to 53.66	≤0.644813

Criterion	Sensitivity	95% CI	Specificity	95% CI	+LR	95% CI	-LR	95% CI
≤2.814840031	85.37	70.8 - 94.4	42.50	31.5 - 54.1	1.48	1.2 - 1.9	0.34	0.2 - 0.8

G

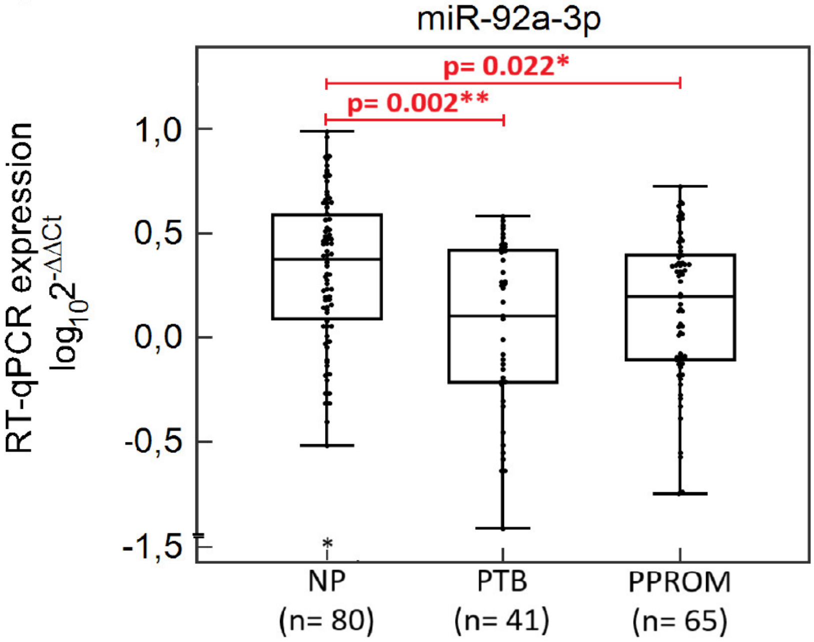

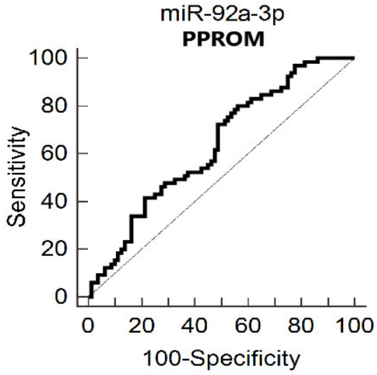

Area under the ROC curve (AUC)	0.634
Standard Error	0.0459
95% Confidence interval	0.550 to 0.712
Significance level P (Area=0.5)	0.0035

Estimated sensitivity at fixed specificity			
Specificity	Sensitivity	95% CI	Criterion
90.00	15.38	4.62 to 30.77	≤0.634862

Criterion	Sensitivity	95% CI	Specificity	95% CI	+LR	95% CI	-LR	95% CI
≤2.720877401	80.00	68.2 - 88.9	43.75	32.7 - 55.3	1.42	1.1 - 1.8	0.46	0.3 - 0.8

H

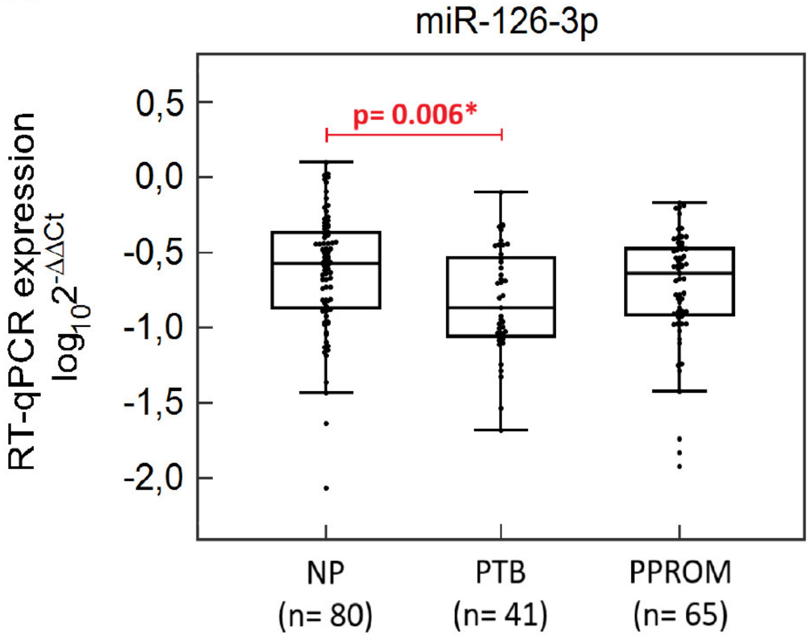

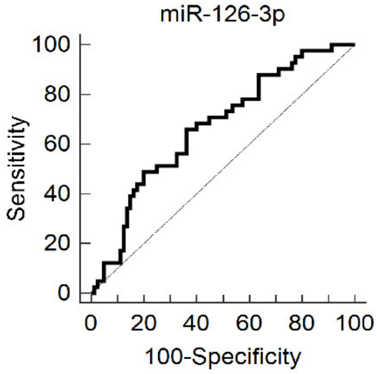

Area under the ROC curve (AUC)	0.666
Standard Error	0.0513
95% Confidence interval	0.574 to 0.749
Significance level P (Area=0.5)	0.0012

Estimated sensitivity at fixed specificity			
Specificity	Sensitivity	95% CI	Criterion
90.00	12.20	0.12 to 36.59	≤0.074792

Criterion	Sensitivity	95% CI	Specificity	95% CI	+LR	95% CI	-LR	95% CI
≤0.209378619	65.85	49.4 - 79.9	63.75	52.2 - 74.2	1.82	1.3 - 2.6	0.54	0.3 - 0.8

I

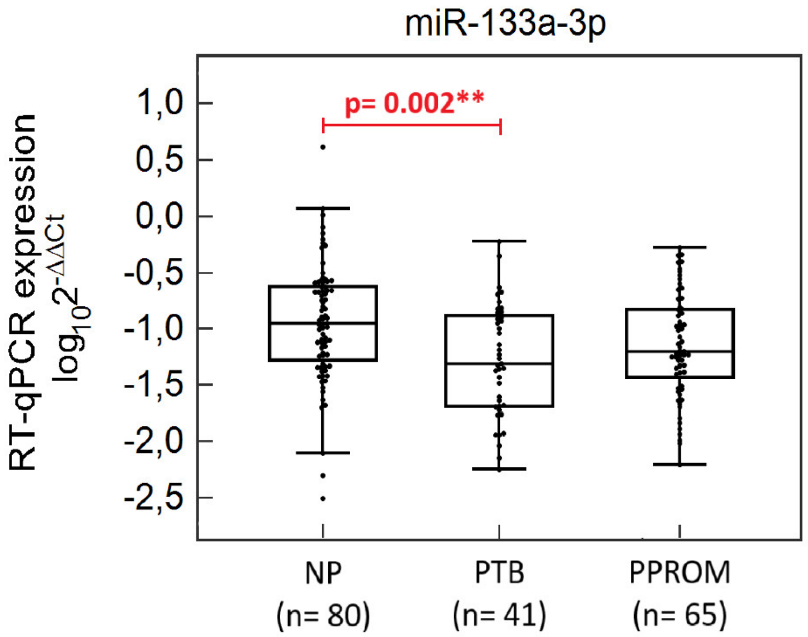

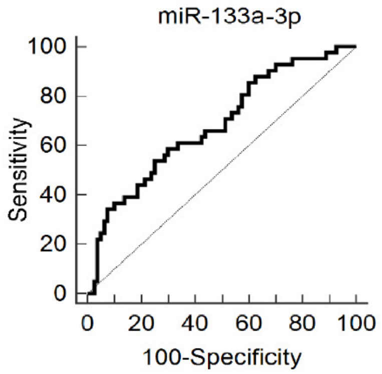

Area under the ROC curve (AUC)	0.682
Standard Error	0.0515
95% Confidence interval	0.591 to 0.764
Significance level P (Area=0.5)	0.0004

Estimated sensitivity at fixed specificity			
Specificity	Sensitivity	95% CI	Criterion
90.00	36.59	19.51 to 52.13	≤0.032746

Criterion	Sensitivity	95% CI	Specificity	95% CI	+LR	95% CI	-LR	95% CI
≤0.054712071	53.66	37.4 - 69.3	75.00	64.1 - 84.0	2.15	1.3 - 3.4	0.62	0.4 - 0.9

J

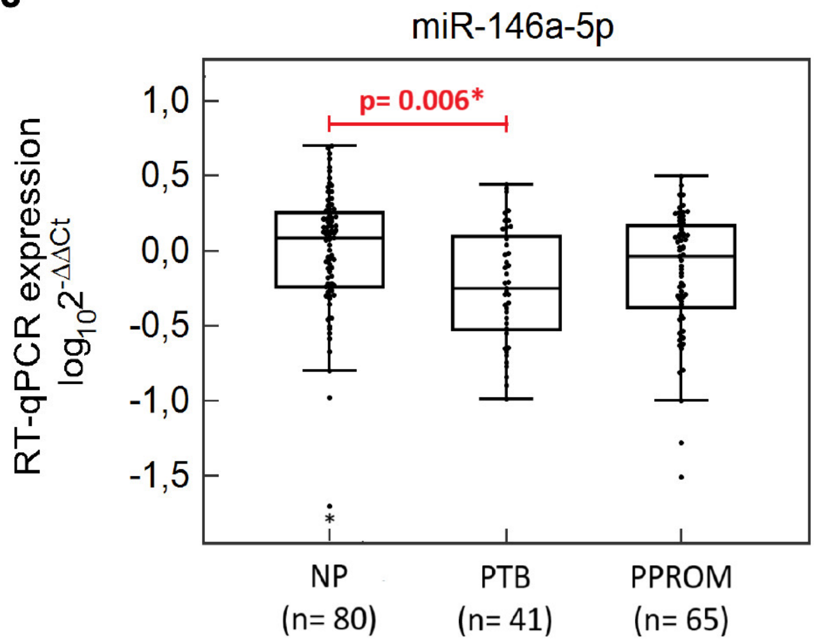

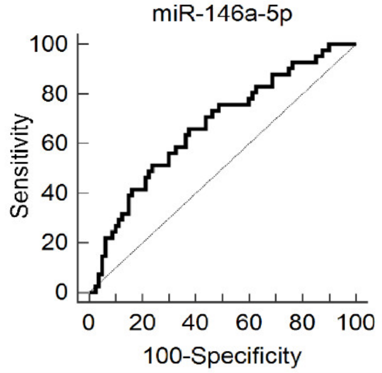

Area under the ROC curve (AUC)	0.669
Standard Error	0.0520
95% Confidence interval	0.578 to 0.752
Significance level P (Area=0.5)	0.0011

Estimated sensitivity at fixed specificity			
Specificity	Sensitivity	95% CI	Criterion
90.00	26.83	7.32 to 46.34	≤0.304521

Criterion	Sensitivity	95% CI	Specificity	95% CI	+LR	95% CI	-LR	95% CI
≤0.840618581	65.85	49.4 - 79.9	62.50	51.0 - 73.1	1.76	1.2 - 2.5	0.55	0.3 - 0.9

K

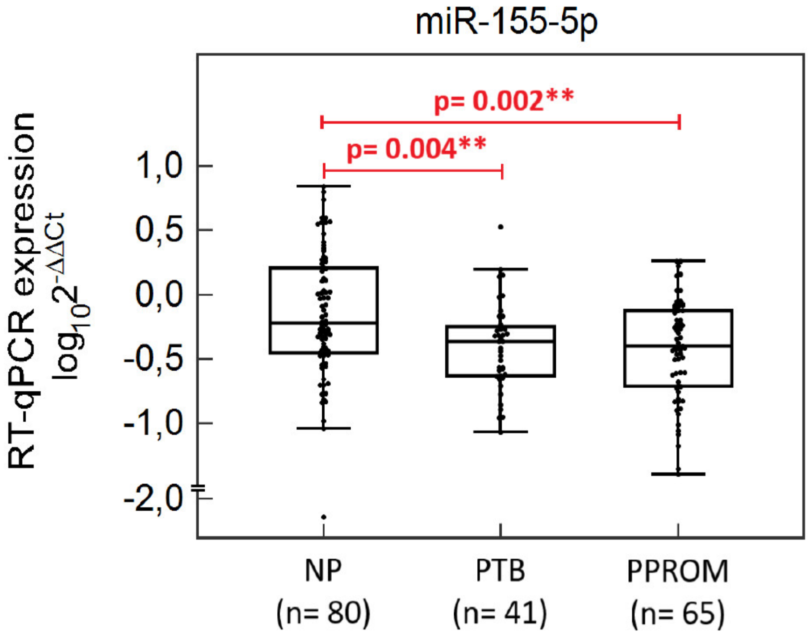

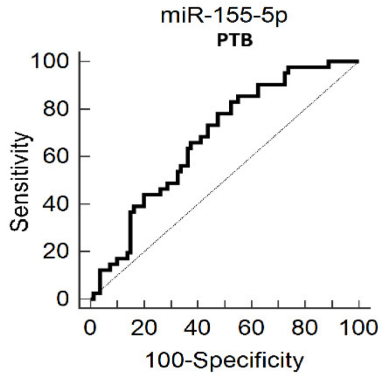

Area under the ROC curve (AUC)	0.677
Standard Error	0.0493
95% Confidence interval	0.586 to 0.759
Significance level P (Area=0.5)	0.0003

Estimated sensitivity at fixed specificity			
Specificity	Sensitivity	95% CI	Criterion
90.00	17.07	4.88 to 46.34	≤0.193078

Criterion	Sensitivity	95% CI	Specificity	95% CI	+LR	95% CI	-LR	95% CI
≤0.585120062	78.05	62.4 - 89.4	52.50	41.0 - 63.8	1.64	1.2 - 2.2	0.42	0.2 - 0.8

L

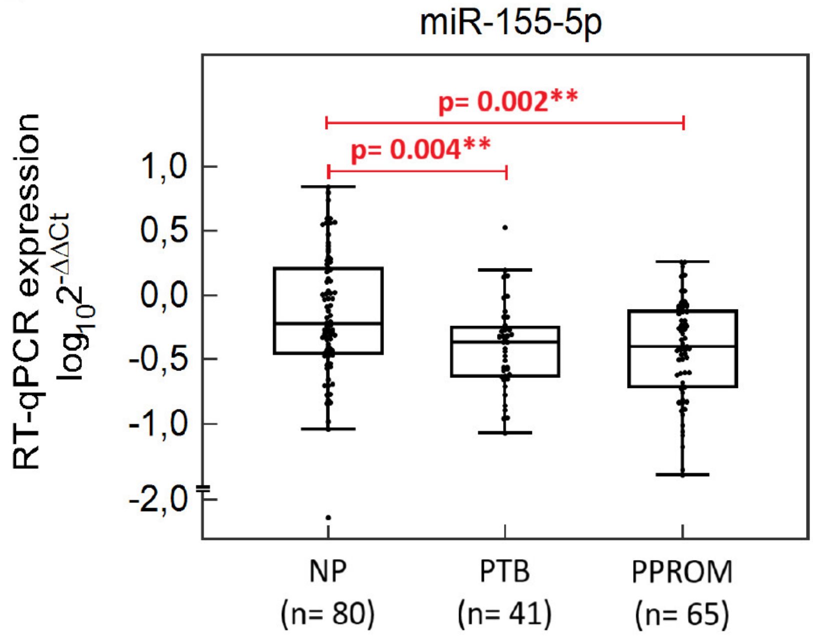

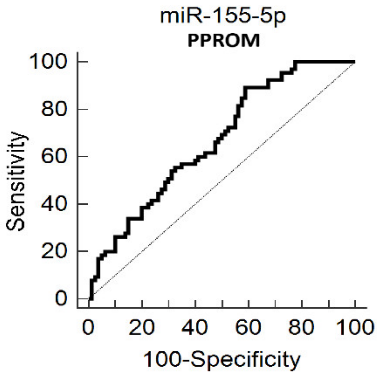

Area under the ROC curve (AUC)	0.667
Standard Error	0.0444
95% Confidence interval	0.584 to 0.743
Significance level P (Area=0.5)	0.0002

Estimated sensitivity at fixed specificity			
Specificity	Sensitivity	95% CI	Criterion
90.00	26.15	15.38 to 43.08	≤0.194107

Criterion	Sensitivity	95% CI	Specificity	95% CI	+LR	95% CI	-LR	95% CI
≤0.891603925	89.23	79.1 - 95.6	41.25	30.4 - 52.8	1.52	1.2 - 1.9	0.26	0.1 - 0.6

M

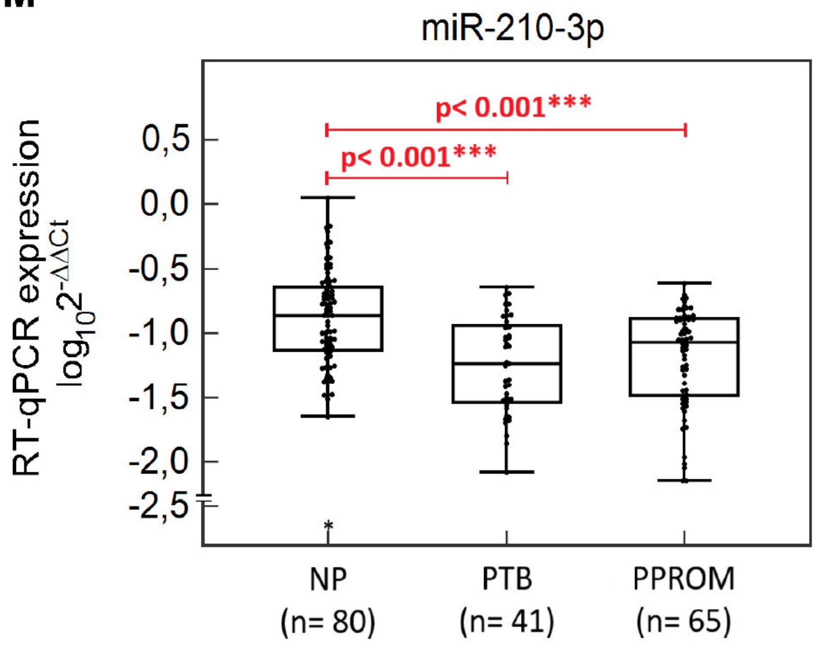

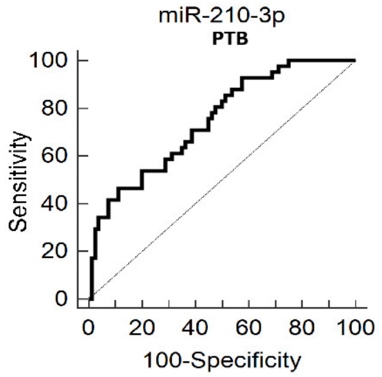

Area under the ROC curve (AUC)	0.746
Standard Error	0.0459
95% Confidence interval	0.659 to 0.821
Significance level P (Area=0.5)	<0.0001

Estimated sensitivity at fixed specificity			
Specificity	Sensitivity	95% CI	Criterion
90.00	41.46	21.95 to 58.54	≤0.041728

Criterion	Sensitivity	95% CI	Specificity	95% CI	+LR	95% CI	-LR	95% CI
≤0.167944775	92.68	80.1 - 98.5	42.50	31.5 - 54.1	1.61	1.3 - 2.0	0.17	0.06 - 0.5

N

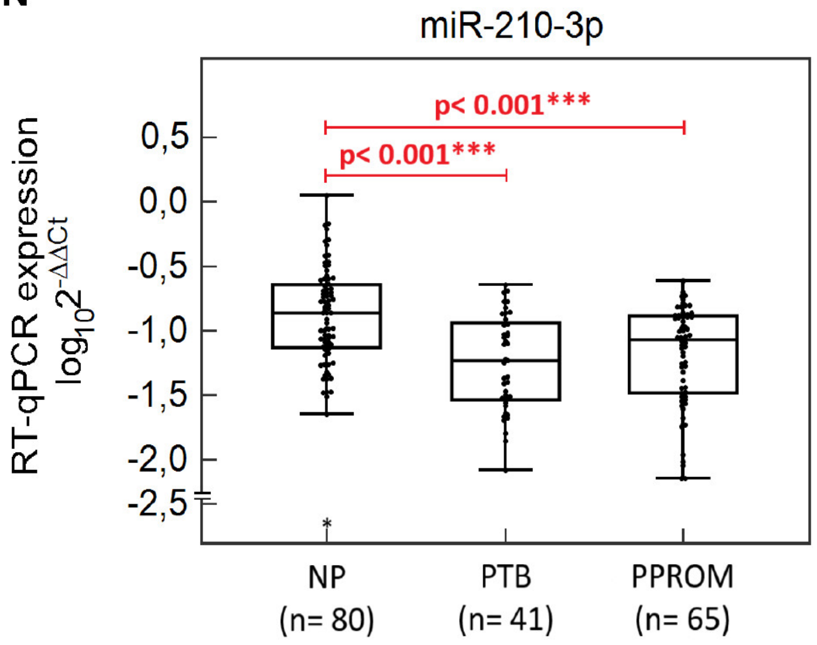

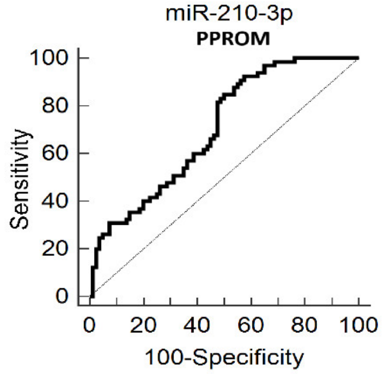

Area under the ROC curve (AUC)	0.702
Standard Error	0.0427
95% Confidence interval	0.620 to 0.775
Significance level P (Area=0.5)	<0.0001

Estimated sensitivity at fixed specificity			
Specificity	Sensitivity	95% CI	Criterion
90.00	30.77	20.00 to 44.62	≤0.041728

Criterion	Sensitivity	95% CI	Specificity	95% CI	+LR	95% CI	-LR	95% CI
≤0.163654728	92.31	83.0 - 97.5	42.50	31.5 - 54.1	1.61	1.3 - 2.0	0.18	0.08 - 0.4

O

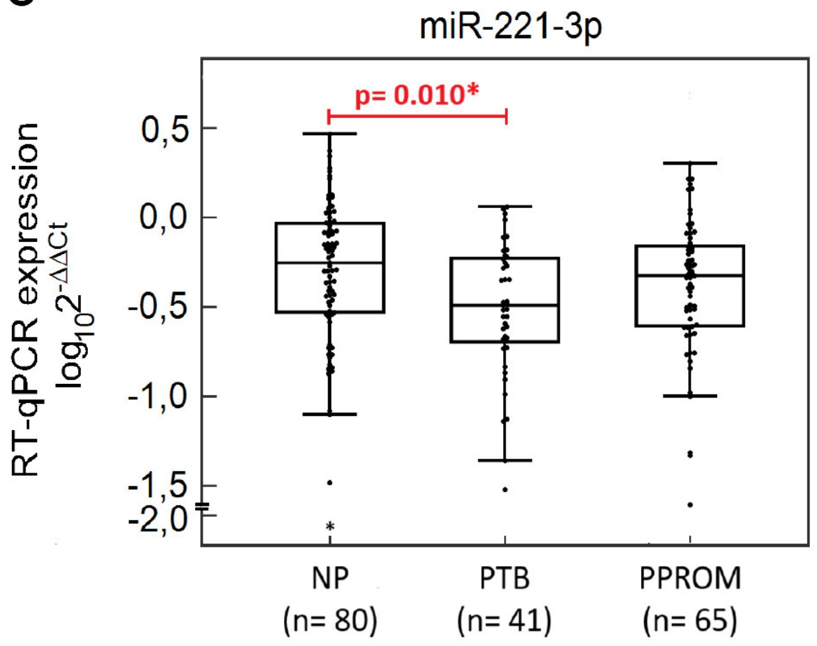

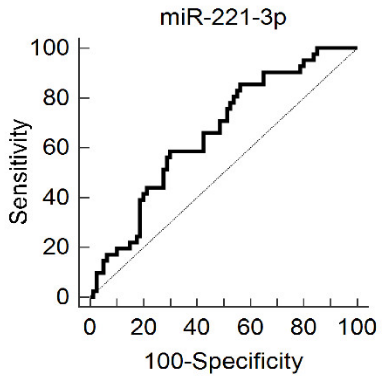

Area under the ROC curve (AUC)	0.660
Standard Error	0.0507
95% Confidence interval	0.568 to 0.744
Significance level P (Area=0.5)	0.0016

Estimated sensitivity at fixed specificity			
Specificity	Sensitivity	95% CI	Criterion
90.00	19.51	7.32 to 39.02	≤0.147589

Criterion	Sensitivity	95% CI	Specificity	95% CI	+LR	95% CI	-LR	95% CI
≤0.656956484	85.37	70.8 - 94.4	43.75	32.7 - 55.3	1.52	1.2 - 1.9	0.33	0.2 - 0.7

P

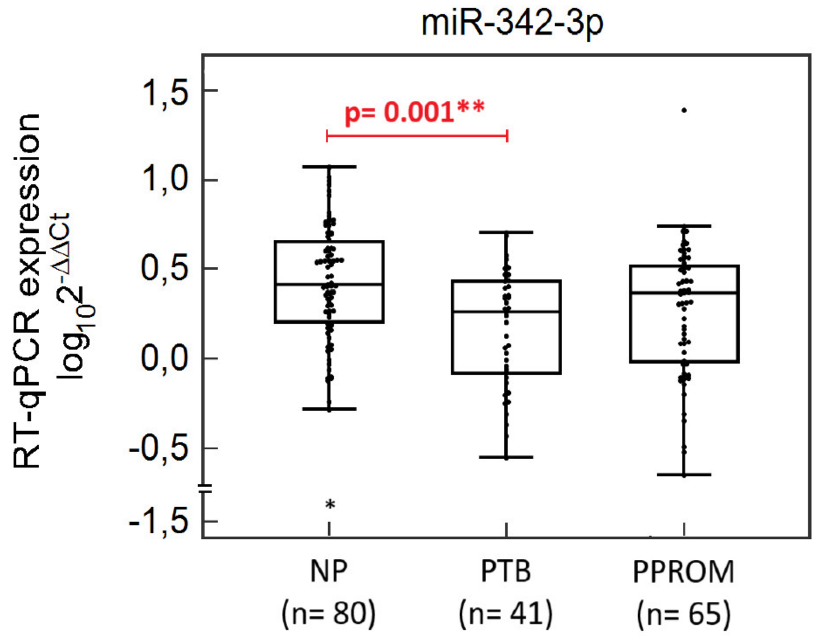

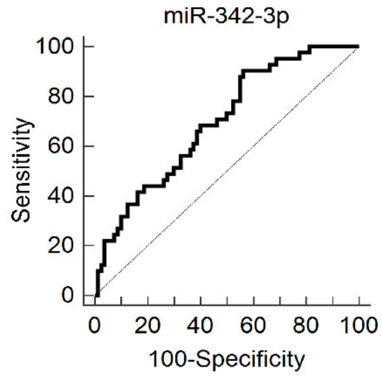

Area under the ROC curve (AUC)	0.696
Standard Error	0.0487
95% Confidence interval	0.606 to 0.776
Significance level P (Area=0.5)	0.0001

Estimated sensitivity at fixed specificity			
Specificity	Sensitivity	95% CI	Criterion
90.00	31.71	14.63 to 50.94	≤0.903942

Criterion	Sensitivity	95% CI	Specificity	95% CI	+LR	95% CI	-LR	95% CI
≤3.281807058	90.24	76.9 - 97.3	43.75	32.7 - 55.3	1.60	1.3 - 2.0	0.22	0.09 - 0.6

Supplementary Figure S5. Gene expression of cardiovascular disease associated microRNAs in peripheral blood leukocytes in early stages of gestation – comparison between NP, PTB and PPROM – statistical significant data after Benjamini-Hochberg correction (results after the Benjamini-Hochberg correction are marked by * for α=0.05, ** for α=0.01, and *** for α=0.001). NP, normal pregnancies; PTB, spontaneous preterm birth; PPROM, preterm prelabor rupture of membranes.
