# Supplementary material for: First Trimester Prediction of Preterm Delivery in the Absence of Other Pregnancy-Related Complications Using Cardiovascular-Disease Associated MicroRNA Biomarkers
Source: Int J Mol Sci. 2022 Apr 1;23(7):3951. doi: 10.3390/ijms23073951 (PMC8999783; doi:10.3390/ijms23073951)
Supplement: Supplementary file 1 [file ijms-23-03951-s001.zip › Supplementary Table S1.pdf]

Supplementary Table S1. Relation between microRNA gene expression and gestational age at birth in patients with preterm delivery (PPROM and PTB).

MicroRNA Gene Expression vs GA at Delivery	ϱ (Spearman's Rank Correlation Coefficient)	*p*-Value
miR-1-3p & GA at delivery	0,137107	0,163103
miR-16-5p & GA at delivery	0,015693	0,873759
miR-17-5p & GA at delivery	0,086921	0,377946
miR-20a-5p & GA at delivery	-0,016886	0,864246
miR-20b-5p & GA at delivery	0,091742	0,351962
miR-21-5p & GA at delivery	0,033435	0,734912
miR-23a-3p & GA at delivery	0,044161	0,654644
miR-24-3p & GA at delivery	-0,017447	0,859785
miR-26a-5p & GA at delivery	0,026071	0,791782
miR-29a-3p & GA at delivery	0,082059	0,405300
miR-92a-3p & GA at delivery	0,073782	0,454454
miR-100-5p & GA at delivery	0,125898	0,200640
miR-103a-3p & GA at delivery	0,106636	0,278941
miR-125b-5p & GA at delivery	0,120600	0,220398
miR-126-3p & GA at delivery	0,083740	0,395712
miR-130b-3p & GA at delivery	0,083787	0,395448
miR-133a-3p & GA at delivery	0,064939	0,510435
miR-143-3p & GA at delivery	0,091379	0,353880
miR-145-5p & GA at delivery	-0,004369	0,964715
miR-146a-5p & GA at delivery	0,024021	0,807823
miR-155-5p & GA at delivery	-0,031676	0,748381
miR-181a-5p & GA at delivery	0,101452	0,303111
miR-195-5p & GA at delivery	0,108058	0,272544
miR-199a-5p & GA at delivery	0,062905	0,523798
miR-210-3p & GA at delivery	0,000192	0,998449
miR-221-3p & GA at delivery	0,068510	0,487414
miR-342-3p & GA at delivery	0,044800	0,649979
miR-499a-5p & GA at delivery	0,005859	0,952700
miR-574-3p & GA at delivery	0,073004	0,459241

GA, gestational age.
